# Synapsins are expressed at neuronal and non-neuronal locations in *Octopus vulgaris*

**DOI:** 10.1038/s41598-019-51899-y

**Published:** 2019-10-28

**Authors:** Federica Maiole, Giulia Tedeschi, Simona Candiani, Luca Maragliano, Fabio Benfenati, Letizia Zullo

**Affiliations:** 1 Center for Micro-BioRobotics & Center for Synaptic Neuroscience and Technology, Istituto Italiano di Tecnologia, Largo Rosanna Benzi 10, 16132 Genova, Italy; 2 Department of Experimental Medicine, University of Genova, viale Benedetto XV, 3, 16132 Genova, Italy; 3 Department of Biomedical Engineering, Laboratory for Fluorescence Dynamics, University of California, Irvine, 92697 CA USA; 4 Laboratory of Developmental Neurobiology, Department of Earth, Environment and Life Sciences, University of Genoa, Viale Benedetto XV 5, 16132 Genoa, Italy; 5 IRCSS Ospedale Policlinico San Martino, Largo Rosanna Benzi 10, 16132 Genova, Italy

**Keywords:** Animal physiology, Non-model organisms

## Abstract

Synapsins are a family of phosphoproteins fundamental to the regulation of neurotransmitter release. They are typically neuron-specific, although recent evidence pointed to their expression in non-neuronal cells where they play a role in exocytosis and vesicle trafficking. In this work, we characterized synapsin transcripts in the invertebrate mollusk *Octopus vulgaris* and present evidence of their expression not only in the brain but also in male and female reproductive organs. We identified three synapsin isoforms phylogenetically correlated to that of other invertebrates and with a modular structure characteristic of mammalian synapsins with a central, highly conserved C domain, important for the protein functions, and less conserved A, B and E domains. Our molecular modeling analysis further provided a solid background for predicting synapsin functional binding to ATP, actin filaments and secretory vesicles. Interestingly, we found that synapsin expression in ovary and testis increased during sexual maturation in cells with a known secretory role, potentially matching the occurrence of a secretion process. This might indicate that its secretory role has evolved across animals according to cell activity in spite of cell identity. We believe that this study may yield insights into the convergent evolution of ubiquitously expressed proteins between vertebrates and invertebrates.

## Introduction

Synapsins are a family of phosphoproteins considered ‘key actors’ in the regulation of neurotransmitter release, for a review see^1^. They are mostly in the nervous system, but also expressed in various cell types where they play a role in exocytosis and vesicle trafficking as well as they do in neurons^2–5^. In a few studies, synapsins have been also identified at the level of the reproductive system, in particular in human spermatozoa and unfertilized zebrafish eggs^4,6^. As synapsins closely associate with synaptic vesicles, their involvement in a wide range of secretory events, including those typical of reproductive organs, can be anticipated.

The existence of non-neuronal functions of synapsins has been attested and studied in a limited number of animal species. Among invertebrates, cephalopods currently represent an important model system for comparative research of conserved, as well as divergent, animal features due to the acquired neural complexity and morphological novelties^7–11^. The recent development of cephalopods’ genomics has provided important tools for a deep understanding of their evolution and comparative investigations with other animal species^7,12^. Since then, several molecular pathways involved in processes, such as morphogenesis, have been found common to vertebrates^8,12–14^.

In this work, we aimed at characterizing the synapsin homologues coded and expressed in the invertebrate *Octopus vulgaris*, with particular attention to their specific localization at the level of brain areas and reproductive organs. We identified three synapsin isoforms phylogenetically correlated to those of invertebrates and with a highly conserved C domain. We present evidence of synapsin expression, not only in the brain, but also in the ovary and testis and further evaluated their spatiotemporal expression at two stages of maturation of the reproductive organs. We found that synapsin expression increases during sexual maturation in cells with a known secretory role and this potentially matches the occurrence of the secretion process. Molecular modeling analysis further provided a solid background for predicting synapsin phosphorylation-dependent binding to ATP, actin filaments and secretory vesicles. These results suggest a role of synapsins in reproductive organ maturation and its possible involvement in vesicle secretion. This protein feature may be conserved across animals and evolved according to the cell function in spite of cell identity.

## Results

### Identification of various isoforms of synapsin in brain, ovary, and testis

**Figure 1:**
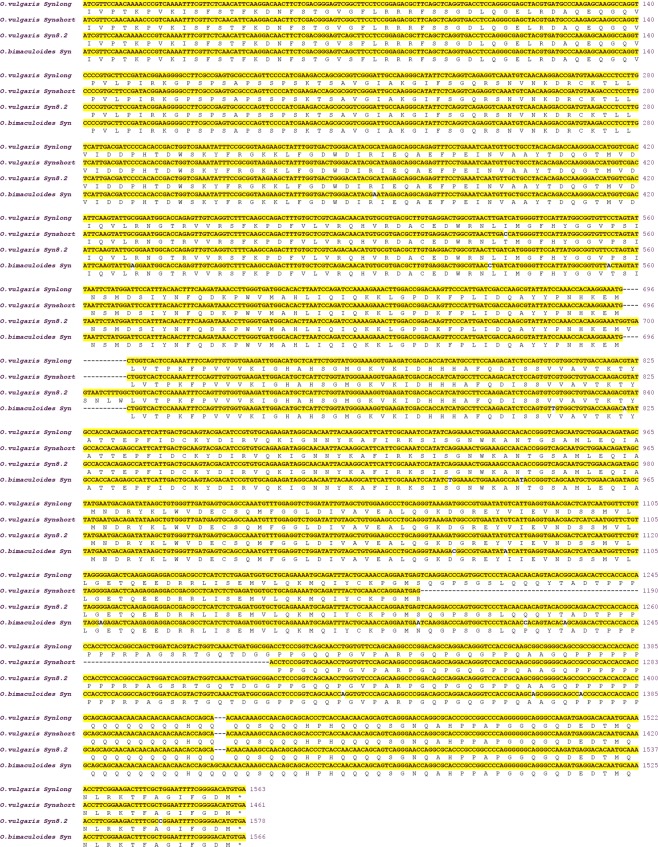
Nucleotide and predicted amino acid sequences of synapsins from *Octopus vulgaris* (identified in the present work) and *Octopus bimaculoides*. Numbers indicate the nucleotide positions (right). The amino acids are reported under the nucleotide sequence. The predicted initiator, ATC (encoding for Isoleucine) is present in all sequences.

**Figure 2:**
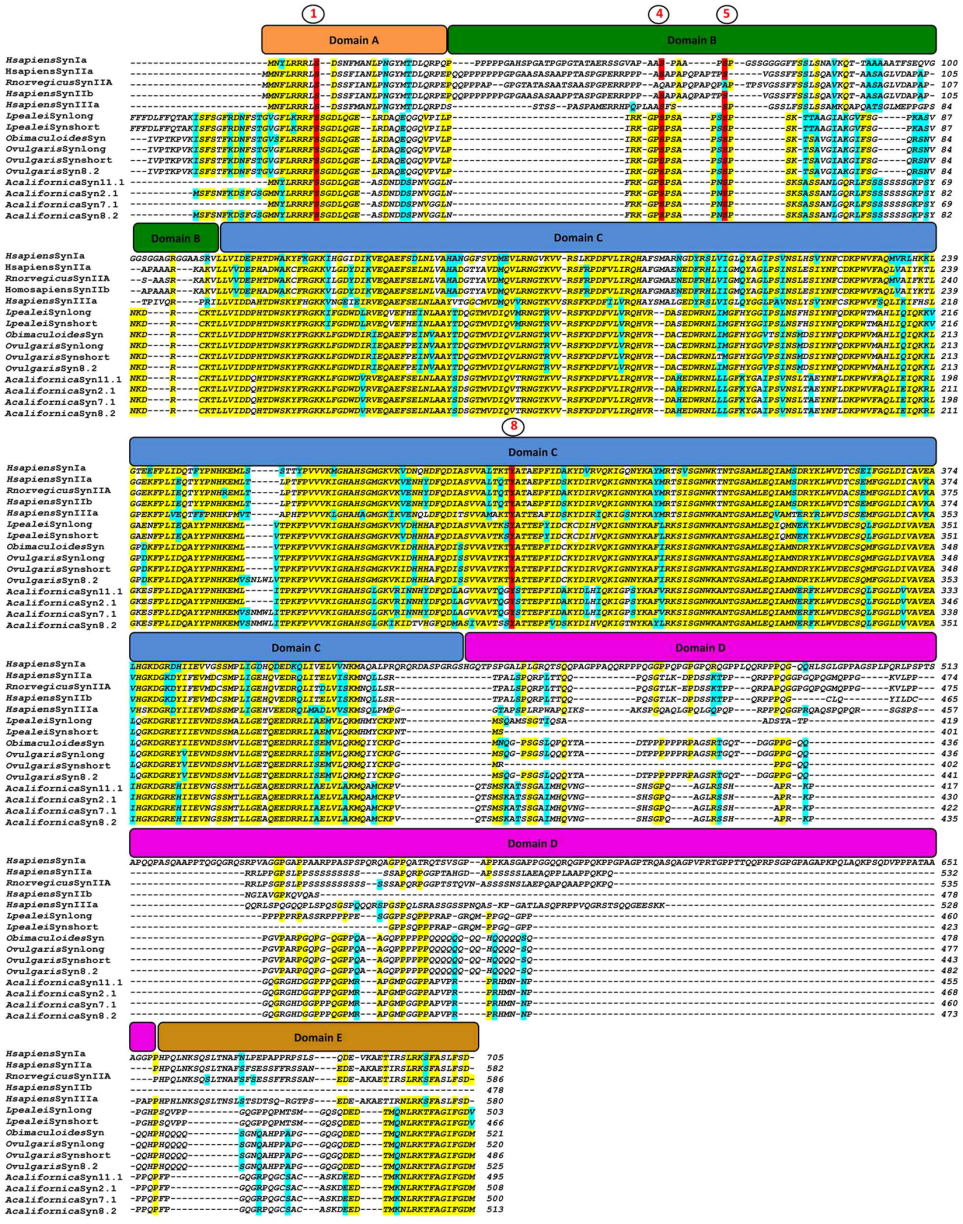
Multiple sequence alignments of synapsin proteins. A and B domains are indicated by orange and green bars. The C-domain is indicated by a blue bar. D and E domains are shown by violet and brown bars, respectively. Identical residues are highlighted in *yellow*, and those conserved in at least 50% of sequences in *light blue*. Conserved phosphorylation sites for PKA and CaMKI/IV (site 1), MAP Kinases (sites 4, 5) and Src kinase (site 8) are shown highlighted in *red*. *O. vulgaris* Syn-long/short sequences are those extracted from the supraesophageal mass (SEM).

Our sequence analysis revealed that *Octopus vulgaris* genome contains a single synapsin gene that is strictly correlated to that of other invertebrates and particularly to the synapsins of other cephalopods such as *Loligo pealei* and *Octopus bimaculoides* (Figs 1 and 2).

Like other invertebrates, *O. vulgaris* possesses synapsin isoforms produced by alternative splicing. PCR experiments on cDNA prepared from brain supraesophageal mass (SEM) yielded two isoforms generated by alternative splicing of the same gene. These two cDNAs result in a long protein, termed *Syn-long*, and a short truncated protein termed *Syn-short* of 521 and 487 amino acids, respectively (Fig. 1, Additional File 1a–g,i–j). Surprisingly, these two transcripts were also isolated from the cDNA of both testis and ovary (Additional file i,j). In these two organs, a third isoform was identified and named *Syn8.2* (Fig. 1, Additional File 1a,f,g,k,l) for the similarity to the *Syn8.2* isoform of *Aplysia californica* (Fig. 2).

The *O. vulgaris* synapsin shows a modular structure characteristic of mammalian synapsins with a central, highly conserved C domain and slightly less conserved A, B and E domains (Fig. 2, see also Additional Files 2 and 3). Domain D shows little primary sequence identity between mammals, mollusks and invertebrates synapsins analyzed to date (Fig. 2, Additional File 3). The short and long synapsin isoforms differ for the absence of 34 amino acids in domain D in the “short” isoform and are similar to those previously identified in *L. pealei*. *O. vulgaris* Syn8.2 protein is characterized by the insertion of a short stretch of 5 amino acids in the central part of domain C (VSNLW). Moreover, *O. vulgaris* Syn 8.2 exhibits some substitutions in domain C that are also present in ApSyn8.2, HpSyn, LpSyn, and mammalian synapsins (Fig. 2). Very few differences in amino acid sequences were identified in the long/short isoforms between ovary/testis and SEM (data not shown). All octopus isoforms contain the E-domain and resemble a-type isoforms of mammalian synapsins. This feature is highly conserved during evolution and supports the functional significance of domain E.

The sequence identity matrix of all synapsin domains in mammals and octopus shows the following features: (i) octopus synapsin A and E domains (20% and 31% identity, respectively) isoforms have a higher similarity with that of human SynIa; (ii) octopus synapsin B domain resembles that of human SynIIIa (43% identity); (iii) octopus synapsin C and D domains have a higher similarity with those of human SynIIa/IIb (respectively 66.9% and 64.7%) and SynIIb (60.2% and 63.6%) (Additional File 3).

**Figure 3:**
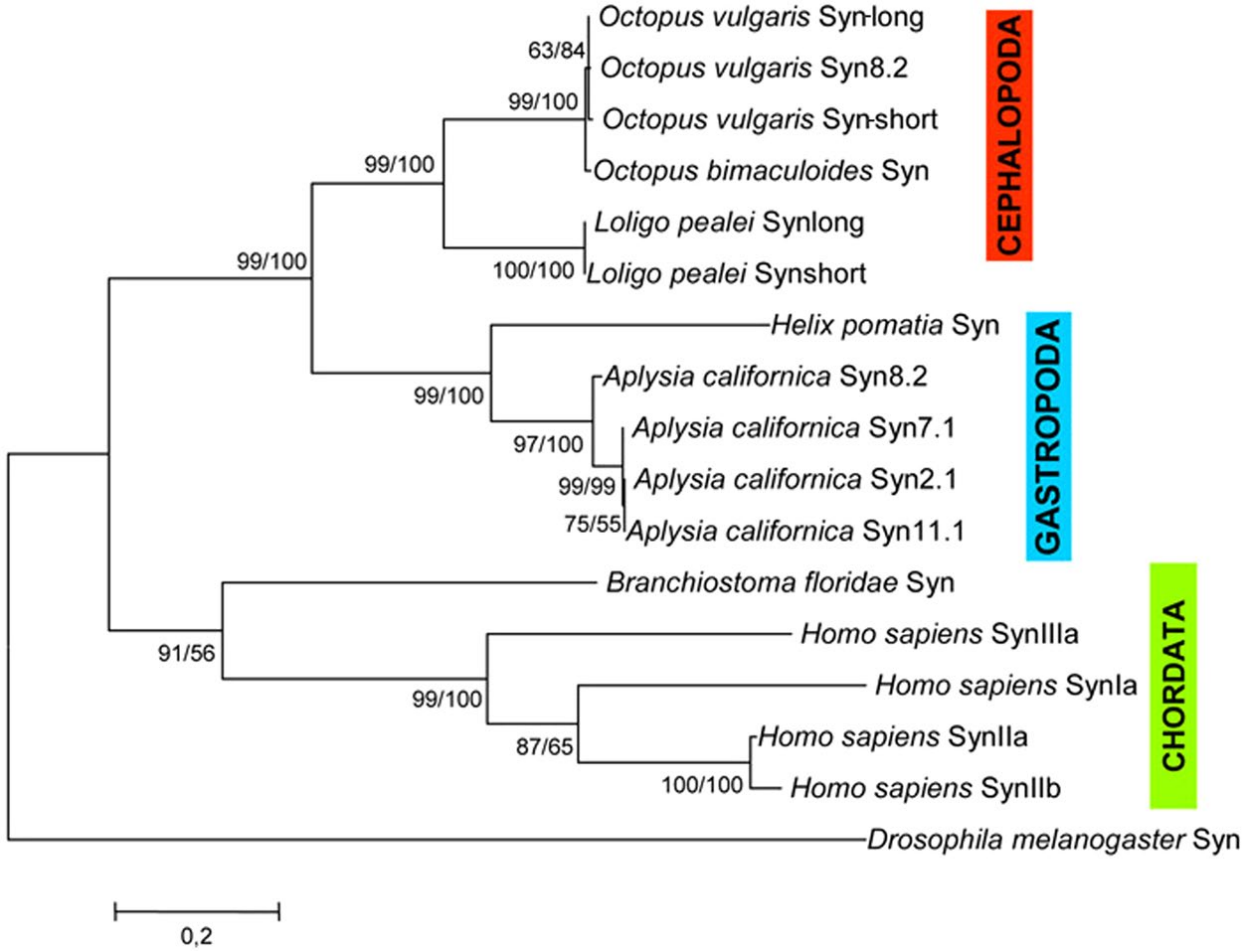
Phylogenetic tree of synapsin proteins in representative metazoan phyla. A rooted phylogenetic tree based on the alignment of the full protein sequences showed in Additional File 4, with *Drosphila melanogaster* synapsin as outgroup. Both NJ and ML methods were used. The ML tree is shown with bootstrap values for ML and NJ analyses (first and second values, respectively); bootstrap confidence limits were obtained through 1000 replicates.

It is also worth noticing that the octopus synapsins start translation codon has turned out to be a non-AUG and in particular an isoleucine (Fig. 1, Additional file h). The presence of a stop translation codon upstream isoleucine supports the hypothesis that the non-AUG codon is used as a start codon. Indeed, this has been reported in *Loligo pealei* and *Octopus bimaculoides* synapsins, where start codons are a phenylalanine and an isoleucine, respectively (Fig. 3, Additional File 1a). The use of non-AUG start codon of translation is an evolutionary conserved phenomenon present from viruses^15,16^ to eukaryotes, and found in both vertebrates and invertebrates^17^. However, to date only synapsins from mollusks are characterized by an unusual start codon. Synapsins are known to be substrates for a variety of protein kinases, and at least nine phosphorylation sites have been described in mammals. Similarly to other mollusks, *O. vulgaris* synapsin shares the following conserved phosphorylation sites with mammals: site 1 (Ser9 in mammalian SynI) for cAMP-dependent protein kinase (PKA), Ca^*2+*^/calmodulin-dependent protein kinase I (CaMKI) and IV (CaMKIV) in the A domain^18^; sites 4 and 5 (Ser62 and Ser67) for mitogen-activated protein kinase (MAPK) in the B domain^19,20^; site 8 (Tyr301) for Src tyrosine kinase in the C domain^21^ (Fig. 2).

### Phylogenetic analysis

Phylogenetic analysis was performed using the Maximum likelihood phylogeny (ML) and close-neighbor-interchange tree (NJ) search methods. Figure 3 shows the ML tree with bootstrap values from both NJ and ML analyses. This phylogenetic tree is based on the alignment of the full protein sequences showed in Additional File 4, with *Drosphila* synapsin as outgroup. In this tree, it is clear that the synapsin of the cephalochordate amphioxus occupies a basal position relative to the three clades of mammals genes: this is consistent with the phylogeny that places the cephalochordate basal to the phylum of the Chordata. In agreement with taxonomic relationships, all mollusks were situated basally to the chordate clade. Furthermore, we observed a clear separation between cephalopod and gastropod synapsins and, among cephalopods, *L. pealei* synapsins were separated from *O. vulgaris* and *O. bimaculoides* synapsins. Our data agree with animal phylogeny and are supported by bootstrap values > 50%.

### *In situ* hybridization on brain, ovary, and testis at various stages of sexual maturation

The reproductive apparatus of animals of both sexes (20 females, 13 males) were first staged, to assess their level of maturation, through gross macroscopic and histologic investigations (see^22^).

**Figure 4:**
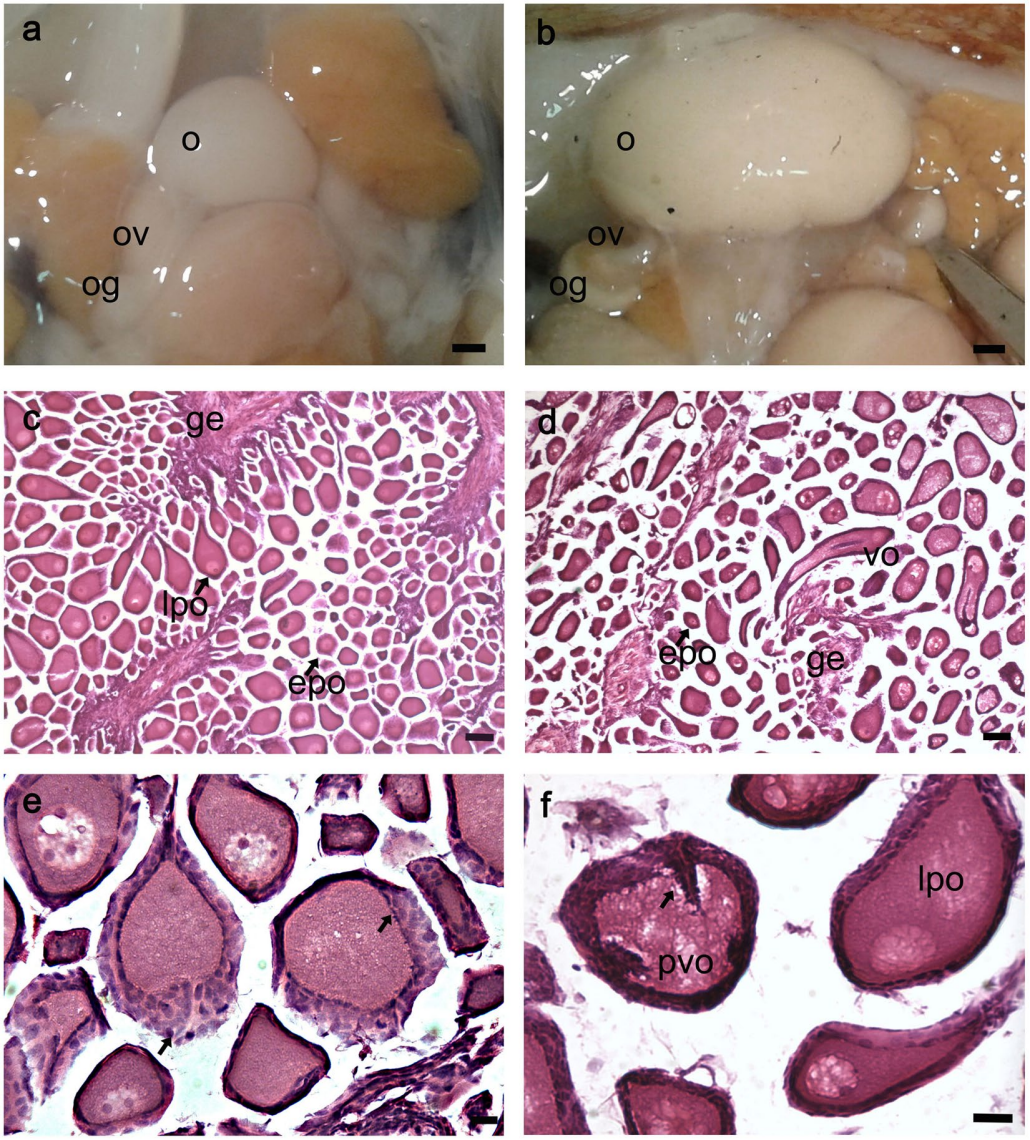
Macroscopic and histological stages of the ovary maturation process. (**a,b**) Ovary gross morphology at an immature (**a**) and mature stage (**b**) (scale bar, 2 mm). (**c**–**f**) Transverse sections of immature (**c,e**) and mature (**d,f**) ovary stained with ematoxylin-eosin at different magnifications (Scale bars, c,d: 100 µm; e,f: 50 µm). Arrows in (**e**) and (**f**) indicate multiple layers of follicular cells without or with invagination, respectively. o: ovary; og: oviducal gland; ov: oviducts; ge: germinal epithelium; epo: early primary oocyte; lpo: late primary oocyte; vo: vitellogenic oocyte; pvo: previtellogenic oocyte.

Samples were grouped into two categories, mature and immature, as described below. Immature female reproductive apparatus (Fig. 4a) presented a small, whitish ovary that did not reach the posterior half of the mantle cavity; the oviducts were visible, the oviducal glands were whitish, round and small. Mature female apparatus (Fig. 4b) consisted of a bigger, yellowish ovary that filled the mantle cavity, visible oviducts and bigger and round oviducal glands, with a white, denticulate apical area and a brownish ring.

Histological analysis of the immature (Fig. 4c) and mature (Fig. 4d) ovaries showed the presence of germinative epithelium, early primary oocytes (round shape, with nucleus and nucleolus visible, surrounded by few follicular cells) and late primary oocytes (nucleus and nucleolus still visible, surrounded by at least one layer of follicular cells) at both stages. At immature stages, several layers of follicular cells (Fig. 4e, arrow) surrounded some of the oocytes. In the mature ovaries, vitellogenic oocytes carrying an elongated shape (Fig. 4d) were also present along with previtellogenic oocytes characterized by invaginating follicular cell layers (Fig. 4f, arrow).

**Figure 5:**
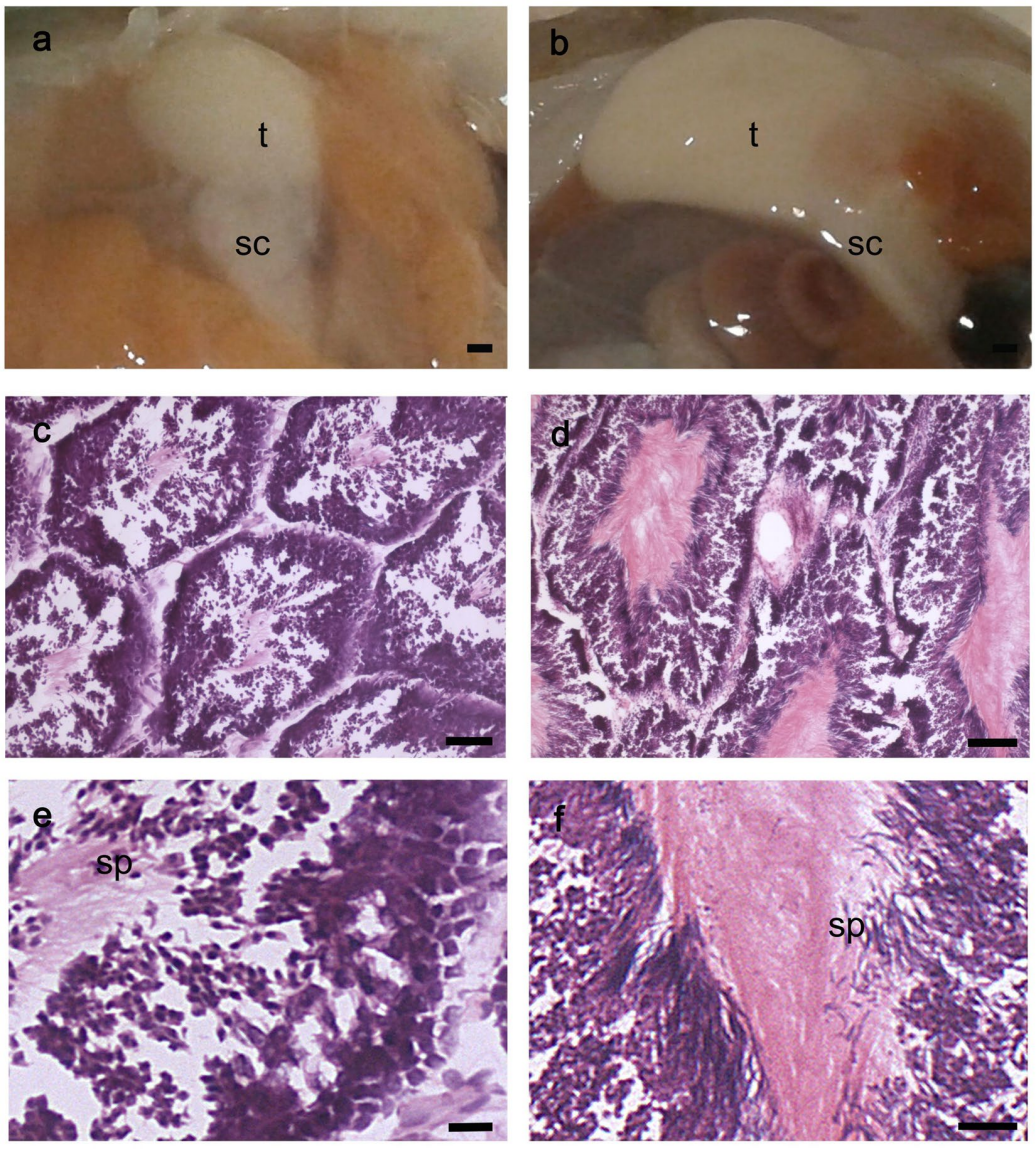
Macroscopic and histological stages of the testis maturation process. Testis gross morphology at an immature (**a**) and mature (**b**) stage (scale bar 2 mm). (**c–f**) Transverse sections of immature (**c,e**) and mature (**d**,**f**) testis stained with ematoxylin-eosin at different magnifications (Scale bar, c–d:100 µm; e: 50 µm; f: 25 µm). t: testis; sc: spermatophoric complex; sp: sperms.

Immature male reproductive apparatus (Fig. 5a) presented a small, round, ivory-white testis and a semi-transparent spermatophoric complex; mature testes (Fig. 5b) were bigger, creamy-white and with a white and well-developed spermatophoric complex. From the histological analysis, the immature testis (Fig. 4c) presented small well-defined seminiferous tubules, while mature testis (Fig. 5d) carried larger tubules with a wider lumen. Spermatogenesis proceeds along the tubule cortex toward its lumen where spermatozoa, with their tails occupying the tubule innermost part, are concentrated. Indeed, in immature tubules, only a few spermatozoa could be observed (Fig. 5e), while in the mature testis a large number of spermatozoa was present (Fig. 5f).

An antisense RNA probe, specific to the common part of the synapsin isoforms, was synthesized and its length verified with gel electrophoresis (see Additional File 5a). Using the ISH technique, we localized synapsin mRNA in samples of ovaries (n = 3) and testis (n = 3) at two distinct stages of sexual maturation.

**Figure 6:**
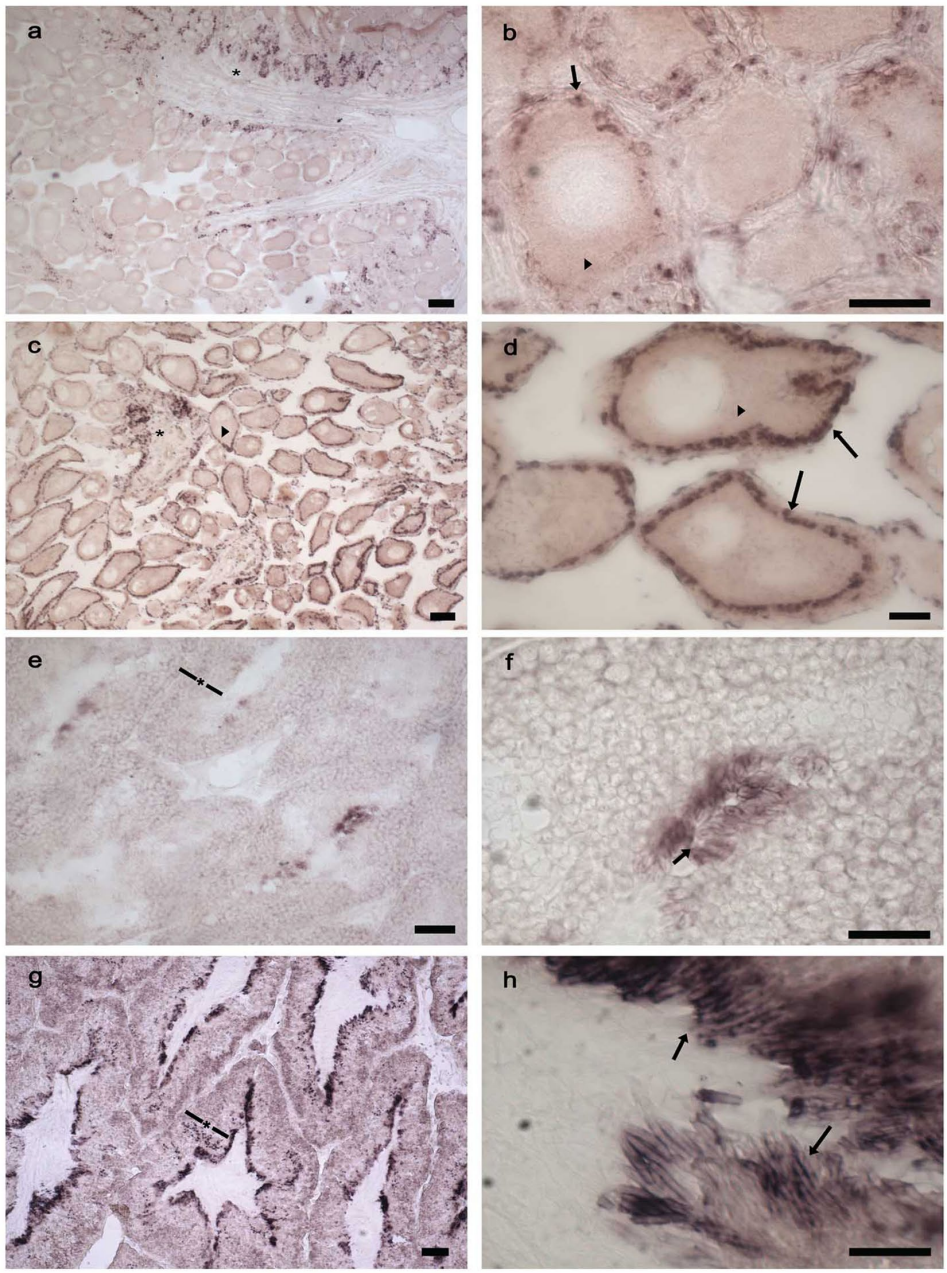
Synapsin *in situ* hybridization on ovary and testis samples at different maturation stages. Representative images of an immature ovary (**a,b**) and of a mature ovary (**c,d**) at two different magnifications. The expression is detected in the germinative epithelium (asterisks) and in follicular cells (arrows), a weak expression is found at the oocyte cytoplasm (arrowheads). (**e**–**h**) Representative images of an immature testis (**e,f**) and of a mature testis (**g,h**) at two different magnifications. Synapsin expression is detected in the tubule cortex (asterisk) and in sperm heads (arrows) scale bar: a,c,e,g: 100 µm; b,d,f: 50 µm; h: 30 µm.

As a positive control, the probe was tested in samples of SEM (Additional File 5b). Synapsin expression localized in the soma of neurons of various lobes, and a high signal was visible in the cortical part of the vertical, subvertical, subfrontal and frontal superior lobes. In immature ovaries, synapsin mainly localized in the germinative epithelium (Fig. 6a, asterisk), in some follicular cells surrounding the primary oocytes (Fig. 6b, arrow) and, with a weaker signal, in the oocyte cytoplasm (Fig. 6b, arrowhead). In the mature ovaries, synapsin expression was still visible in the germinative epithelium (Fig. 6c, asterisk) and in the oocyte cytoplasm (Fig. 6c,d, arrowheads), but was stronger in follicular cells surrounding the oocytes (Fig. 6d, arrows). When more layers of follicular cells were present, the most positive cells localized in the inner layers facing the oocyte. In the immature testes, synapsin was expressed at the tubules cortex (Fig. 6e, asterisk) and concentrated around the sperm heads (at the border of the lumen) (Fig. 6f, arrow). In the mature testes, a stronger signal was found in the entire tubules cortex (Fig. 6g, asterisk) and around the heads of spermatozoa (Fig. 6h, arrows).

### Localization of synapsin protein

**Figure 7:**
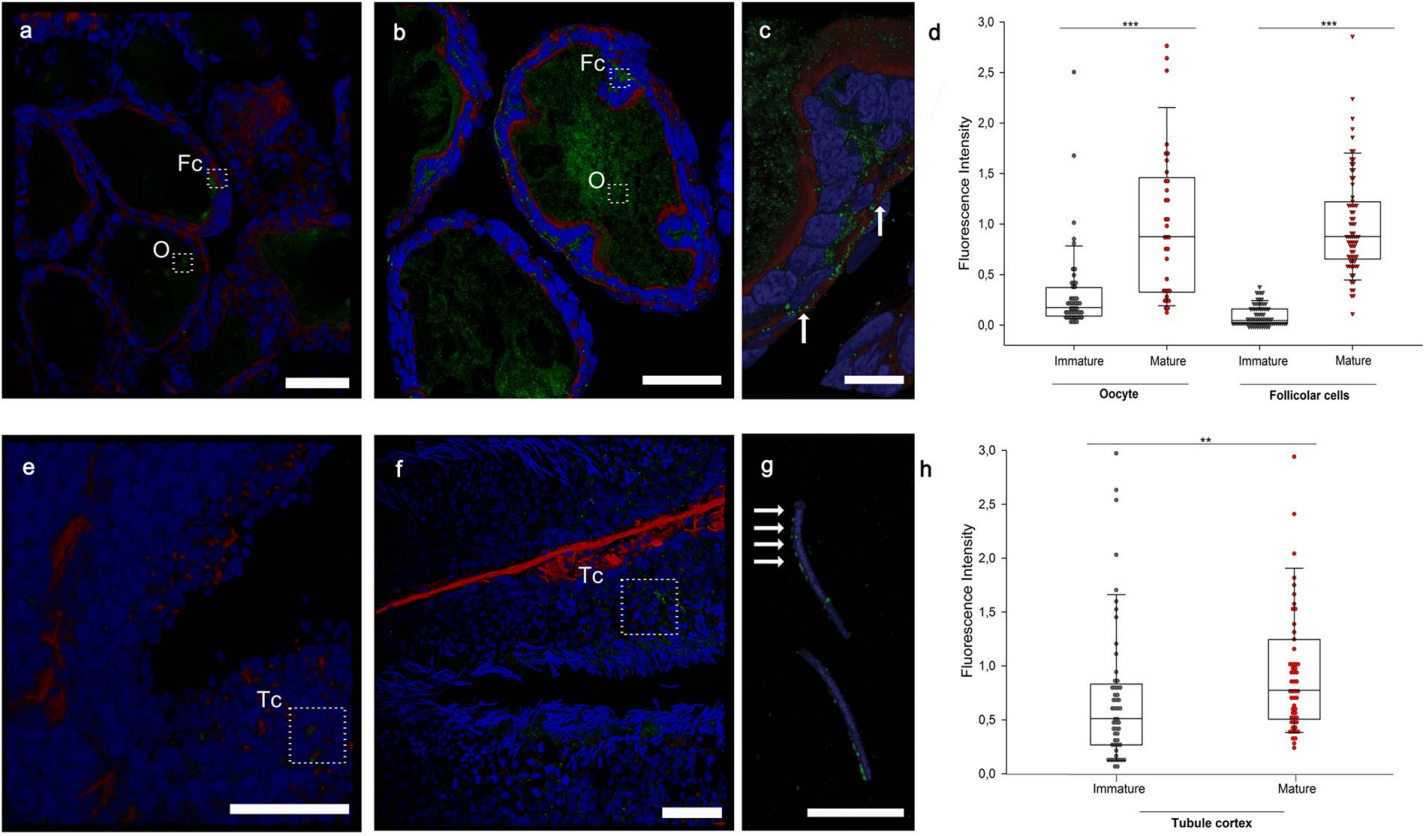
Synapsin immunolocalization during gonadal maturation. (**a**,**b**) Confocal images of an immature (**a**) and a mature (**b**) ovary stained for synapsin (green), F-actin (phalloidin, red) and Hoechst (blue). Representative areas used for fluorescence intensity quantification from oocytes (O) and follicular cells (Fc) are shown in rectangles. (**c**) Higher magnification of a mature ovary where a clear synapsin signal is present at the level of the follicular cells with a punctate pattern **(c**, arrows**)**. (**d**) Quantification of synapsin fluorescence intensity in the cytoplasm of the oocyte and in follicular cells (*t*-test, ***p < 0.001). (**e,f**) Confocal images of immature and a mature testes, respectively. Representative areas used for fluorescence intensity quantification from tubule cortex (Tc) are shown in rectangles. (**g**) Higher magnification of sperm heads in the mature testis. Note the clear punctate perinuclear localization of synapsin (arrows). (**h**) Quantification of synapsin fluorescence intensity in the tubule cortex of immature and mature testis (*t*-test, **p < 0.01) (Scale bars, a,b,e,f: 50 µm; c,g: 10 µm).

To determine synapsin localization and its expression level during sexual maturation in both sexes, an immunostaining assay was performed at mature and immature stages using the anti-synapsin E domain antibody (G-304) previously validated by immunoblot on squid optic lobe extract^23,24^. Synapsin was observed in the oocyte cytoplasm with a widespread distribution and in follicular cells with a punctate localization in both immature (Fig. 7a) and mature (Fig. 7b,c) ovaries. The quantification of synapsin immunofluorescence at both sites showed that its expression significantly increased during the maturation process (Fig. 7d, *t*-test p < 0.001). In immature (Fig. 7e) and mature (Fig. 7f,g) testis, synapsin was expressed along the tubule cortex and presented a punctate pattern around the sperm heads, surrounding the nuclei. Octopus spermatozoa at this stage of development have an elongated shape with most of the cytoplasmic space at the sperm head occupied by the nucleus. Quantification of synapsin immunofluorescence revealed that synapsin was enriched in the cortex of seminiferous tubules at mature stages (Fig. 7h, *t*-test p < 0.01).

### Synapsin modeling and active sites conservation

**Figure 8:**
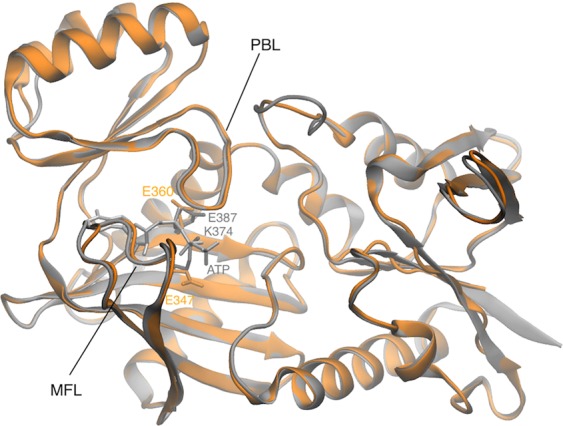
Structural model of *O. vulgaris* synapsin C domain. *O. vulgaris* synapsin C domain (orange) is superimposed to the crystal structure of *R. norvegicus* synapsin II C domain (gray). Charged residues relevant for ATP binding in *R. norvegicus* and the corresponding ones in *O. vulgaris* are represented as sticks. The multi-functional (MFL) and phosphate binding (PBL) loops are also indicated.

We used the I-TASSER online software to generate a structural model of the *O. vulgaris* synapsin C domain (residues X to Y)^25^. The threading algorithm identified the C domains of *R. norvegicus* synapsin II (PDB code 1i7l) and I (PDB code 1pk8) as the best templates for *O. vulgaris* synapsin C domain. The two template domains show a very high sequence and structural identity, with a Root Mean Square Deviation (RMSD) between them of 0.8 Å. The *O. vulgaris* and *R. norvegicus* C domain sequences have 65% identity, and the alignment used to predict the structure extended over 95% of the residues. The best ranking I-TASSER model of *O. vulgaris* synapsin C domain is shown in Fig. 8, superimposed to the *R. norvegicus* synapsin II C domain. The model had C-score 0.70 (74% of optimal value) and TM-score 0.81 (60% above correct topology threshold), highlighting the very good quality of the predicted structure (see Methods for a brief explanation of these parameters). The RMSD between the model and the C domains of *R. norvegicus* synapsins II and I were 0.47 and 0.7 Å, respectively, values typical of very similar folded conformations. Indeed, as can be seen in Fig. 8, the model’s backbone is nearly identical to *R. norvegicus* synapsin II C domain, and the structure displays all the characteristic features of synapsin C domains that have been shown to mediate ATP binding. These include charged residues near the (putative) ATP binding site, the multifunctional (MFL) and the phosphate-binding loop (PBL).

However, *O. vulgaris* synapsin C domain has two glutamate residues at the ATP binding site (Glu347 and Glu360), similarly to synapsin I (where the corresponding residues are Glu373 and Glu386) and opposite to synapsin II and III, where the first glutamate is substituted by a lysine (Lys374 and Lys352, respectively). This inversion of amino acid charge has been shown to be responsible for the different regulation exerted by Ca^2+^ on ATP-binding to synapsins I, II and III^26–28^.

## Discussion

Synapsins are evolutionarily conserved phosphoproteins primarily expressed in the brain and fundamental to the regulation of neurotransmitter release. However, recent studies reported their presence in non-neuronal cell types where they play a role in exocytosis and vesicle trafficking^2–5^. Interestingly, an increasing number of molecules considered neuron-specific, such as Tsga10, tau protein, and cadherin, have been found in both nervous tissue and reproductive organs^29–31^.

Although this might seem strange, one should consider that several processes occurring in cells of the reproductive organs during sexual maturation recall somehow the life of a neuron. Starting from their active division, differentiation, and migration, at the beginning of their development, they end acquiring a remarkable secretory function that is at the basis of neurotransmission. All these processes require a well-coordinated rearrangement of the intracellular and extracellular tissue scaffolding and the mobilization of vesicles across cytoskeletal elements mediated by interacting proteins. In particular, the last function is exploited through synapsin ‘sandwich’ association between the surface of synaptic vesicles and cytoskeletal filaments^5,23,32–37^. This is possible by the cooperative functions of domains specific for actin filament and membrane phospholipids binding. Several functional domains, named from A to J, can be differently assembled and give rise to alternative transcripts. The majority of these domains are highly conserved across vertebrates and even invertebrates^24,38,39^. Domain C is the core of synapsin function and has a crucial role in many of the conserved functions such as actin and phospholipid interaction, ATP binding and phosphorylation^39^. Domains A, C, and E characterize the prototypical invertebrate synapsins and share high similarity with vertebrates. Domain B is known to be a conserved linker connecting domains A and C. Its specific functions have not been fully described but the presence of two phosphorylation sites for the mitogen-activated protein kinase (MAPK)/extracellular signal-regulated kinase (Erk) suggests a regulation of synapsin function by this signaling pathway^19,40^. These sites are conserved in octopus synapsin as shown in Fig. 2 (sites 4, 5) thus pointing toward the existence of a similar regulatory mechanism of synapsin function. Downstream the MAP Kinases sites, mammalian synapsin domain B contains an evolutionary conserved amphipathic lipid packing sensor motif (ALPS) that works as a sensor of membrane curvature and enhances the binding of synapsins to synaptic vesicles^41^. ALPS are able to insert into curved membranes and have been identified in several proteins including Syn I, Syn II and Syn III^41,42^. This region is not conserved in octopus synapsin thus suggesting that protein-targeting may be controlled through mechanisms different from that found in vertebrates. Indeed, the interaction between domain B and C is considered important for the synapsin correct localization^43^.

When seen from an evolutionary perspective, the acquisition of domains meets the increase in nervous system complexity. Given the highly developed nervous system of cephalopods, these animals might represent a valuable biological model to study synapsin evolution and function. The recent advances in cephalopod genomics have boosted many fields in cephalopod research; it is now clear that, in spite of their evolutionary divergence, several common principles of morphogenesis and physiological processes exist between vertebrates and invertebrates^7,8,14^.

In this study, we characterized the synapsin isoforms coded and expressed in *Octopus vulgaris* brain and reproductive organs. We first identified three synapsin isoforms, phylogenetically correlated to that of invertebrates; all contained an E domain, a highly conserved C domain and less conserved A, B and E domains.

Octopus synapsin sequences, as in other mollusks, are characterized by non-AUG canonical start codon. It is well known that translation can start at non-AUG codons across eukaryotes^20–22^, but no data currently supports the existence of a non-canonical AUG in vertebrates and mammals synapsins. Despite this, our study reveals novel insights into the use of non-AUG translation to expand the functional coding potential of octopus genome.

In the current work, we present evidence of synapsin expression in brain, ovary, and testis and show its increase during sexual maturation in cells with a known secretory role. This potentially matches the occurrence of a secretion process in reproductive organs, such as the release of vitellogenic granules from follicle cells to the oocyte, and the sperm heads acrosomal reaction. Interestingly, one other large family of membrane receptor protein, the G protein-coupled receptor (GPCR) is known to act at the interphase between hormonal stimulation and reproductive organ response thus controlling sexual maturation.

It has already been shown in other invertebrates, that during female reproduction, vitellogenin (Vg) gene expression and the uptake of Vg or yolk protein into the oocyte is dependent on the correct expression and function of GPCRs^44^. Both octopus ovary and testis are enriched in several GPCR-like adhesion receptor families^7^, thus opening the possibility that a secretory machinery possibly involving GPCRs is activated in these organs during maturation and functions. Expression of GPCR proteins has been assessed also in male reproductive system in a number of organisms including mammals. GPCRs have been proven to be associated to sperm physiology^45,46^ and, in particular, to acrosome reaction through a variety of signaling pathway^45,46^.

The acrosome reaction is a stimulus-induced exocytosis process activated by an increase in cytoplasmic Ca^2+^, just like Ca^2+^-dependent neurotransmitter release. This phenomenon occurs also in cephalopods where a calcium-mediated mechanism induces acrosome reaction through the bulging, vesiculation, and dehiscence of the plasma membrane around the acrosome and the nucleus. It is worth notice that this process resembles the acrosome reaction studied in other species^47,48^. Vesicles localization around the sperm nucleus observed in these studies is in line with the perinuclear localization of synapsin found in our samples of testis, although further functional investigations are needed to clarify the specific function of synapsins in sperm activity.

Interestingly, our sequence and molecular modeling analysis provide a solid background for predicting synapsin Ca^2+^-dependent functional binding to ATP, actin filaments and secretory vesicles in a phosphorylation-dependent manner. The available crystal structure of synapsin isoforms from different species revealed that the octopus synapsin C domain is structurally homologous to a family of ATP-binding enzymes^26,27^. Synapsins are known to bind ATP with high affinity although with a differential regulation by Ca^2+^. In particular, while Ca^2+^ activates ATP binding to synapsin I, it does not affect ATP binding to synapsin II, and inhibits ATP binding to synapsin III^26,27^. In synapsin I, the Ca^2+^ requirement for ATP binding is mediated by an evolutionarily conserved glutamate residue (Glu373) at a position where synapsins II and III contain a lysine residue (Lys374 and Lys352, respectively). Similar to synapsin I, octopus synapsin displays a glutamate residue (Glu347) in that position, pointing to a Ca^2+^-regulated ATP binding.

The presence of synapsin in non-neuronal tissues may be the starting point for explaining the role of synapsin-like molecules in simple organisms lacking a nervous system, such as protozoa, where a molecule similar to synapsin, formed only by the C domain, was discovered. In fact, from a functional viewpoint, the C domain is important for both the binding to ATP molecules and the cytoskeletal components of actin.

Interestingly, the presence in the octopus genome of other genes coding for molecules involved in cell-cell interaction and signaling to the actin cytoskeleton, such as cadherins, and particularly their expression in reproductive organs has been already assessed^7,12^.

Taken together our results point toward the possibility that, in the octopus, a similar regulatory mechanism is involved in the process of vesicle secretion occurring in phenotypically different cells. Although the tissue-specific role of many ubiquitously expressed protein is still unknown in many animal species, recent evidence points toward the conservation of their function in the different cell types^49^. Within this framework, our study might yield insights into their convergent evolution and maintenance of molecular and functional conserved roles across metazoans.

## Methods

### Animal collection and RNA preparation

Specimens of both sexes were collected from local anglers of the ligurian coast of Italy. All our research conformed to the ethical principles of the three Rs (replacement, reduction and refinement) and of minimizing animal suffering, following the Directive 2010/63/EU (Italian D. Lgs. n. 26/2014) and the guidelines from Fiorito *et al*.^50^. All experimental procedures were previously approved by the local Ethical Committee (OPBA (Organismo preposto al benessere degli animali) of the IRCCS (Istituto di Ricovero e Cura a Carattere Scientifico) Ospedale Policlinico San Martino, Genova, Italy) and by the Italian Ministry of Health (authorization n. 1111/2016-PR) and all experiments were performed in accordance with relevant guidelines and regulations.

Animals were anesthetized in cold seawater supplemented with 1% ethanol and 55 mM MgCl_2_. Brain supraesophageal mass (SEM), ovary and testis were collected and frozen for RNA extraction or fixed for *in situ* hybridization (ISH) and immunofluorescence. Total RNA from these samples was extracted using the RNeasy Microarray Tissue Mini Kit (QIAGEN). Following extraction, RNA was treated with RNAse-free DNAse according to the manufacturer’s recommendations to digest contaminating genomic DNA. For each sample, 1 μg of total RNA was retrotranscribed with Superscript III First Strand Synthesis SuperMix (Invitrogen) following the company’s instructions.

### Cloning and sequencing

A blast search by using the available genome of *Octopus bimaculoides* (JGI-Metazome: https://metazome.jgi.doe.gov/pz/portal.html) was used to identify homologs of synapsin. A partial sequence resembling the synapsin sequence from *O. bimaculoides* was identified (JGI: LOC106879677 and NCBI: XM_014929352.1) and used to construct partially overlapping primers to isolate a clone from *O. vulgaris* (see Additional File 1b–d). Subsequently, the genomic locus of *O. bimaculoides* was further analyzed to obtain the entire coding sequence (Additional File 1a). In particular, the clone Ocbimv22001810m (Location: Scaffold 111264:29688:65727) from JGI-Metazome was used to identify the putative start codon. Amplicons from *O. vulgaris* were amplified by PCR from SEM, ovary and testis cDNAs. PCR experiments were carried out in a 25 μl reaction mixture using the DreamTaq DNA Polymerase according to the manufacturer’s instructions (Thermo Scientific). PCR experiments gave the expected synapsin sequence together with other synapsin isoforms (Additional File 1e). The PCR products were directly cloned using pGEM®-T Easy Vector System (Promega, Milan, Italy) and then sequenced using a genetic analyzer 3130 (Applied Biosystems Life Technologies, Milan, Italy). The coding sequence for the *O. vulgaris* synapsin mRNA short and long isoforms, as well as the alternative isoform isolated in ovary and testis (named *Syn8.2*), were deposited in NCBI under the following accession numbers: *Syn-long:* KY768851; *Syn-short*: KY768852; *Syn8.2*: KY768853.

### Sequence and phylogenetic analysis

The Vector NTI Suite (version 9.0, Informax, North Bethesda, MD, USA) software package was used for sequence analysis. Protein sequences of synapsin from several organisms were aligned using ClustalW in the MEGALIGN program from Lasergene (DNASTAR, Madison, WI).

Accession numbers of sequences used in this study and the multi-alignment used to reconstruct the phylogenetic tree are reported in the Additional File 2, respectively.

Comparison between synapsin domains was performed by sequence identity matrix using GeneWorks (Clustal W method with PAM250 weighting and identical gap costs) (Additional File 3).

The evolutionary history was then inferred using maximum likelihood (ML) and neighbor-joining (NJ) methods in MEGA 6.0^51^ using the pairwise deletion option and 1000 bootstraps. The alignment used to generate Fig. 3 is shown in Additional File 4. The most appropriate evolutionary model for phylogenetic inference was identified using Model Selection implemented in MEGA, selecting models with the lowest BIC scores. The JTT model with Gamma distribution (JTT + G) proved the best fit, and an evolutionary history was then inferred using the ML method based on the JTT matrix-based model.

The rate of variation among sites was modeled with a gamma distribution with shape parameter = 1.1358, in an analysis involving 17 amino acid sequences. For the NJ method, evolutionary distances were computed using the JTT matrix-based method and are expressed as numbers of amino acid substitutions per site. Bootstrap confidence limits were obtained by 1000 replicates in both ML and NJ analysis. Tree files were viewed by using MEGA 6.0.

### *In situ* hybridization and histology

A digoxigenin-labeled antisense riboprobe for synapsin was synthesized from cDNAs cloned in the pGEM®-T Easy Vector (Promega) following the manufacturer’s protocol (RNA labeling kit, Roche Applied Science, Monza, Italy). The length and quality of the DIG-labeled RNA synapsin probe was controlled on an agarose gel. According to manufacturers, 1 μg of probe was added to 3 volumes of RNA sample loading buffer (Sigma-Aldrich) heated to 65 °C for 10 minutes, and then chilled on ice. The probe was loaded onto a TAE/1% agarose/sybrSafe gel next to 1 kb ladder (ThermoFisher Scientific) and run at 80 V.

One band at the expected length of ≈810 nt is clearly visible (see Additional File 5a). The probe was a fragment of ~810 nucleotides complementary to the common part of long and short synapsin isoforms. *In situ* hybridization (ISH) was carried out on *O. vulgaris* SEM (see Additional File 5b), ovary and testis 20 μm-frozen sections as described by^14,52^, hybridized overnight at 60 °C. Sections were post-fixed in 4% paraformaldehyde (PFA) and mounted with Mowiol. Parallel sections were stained with hematoxylin and eosin (Sigma-Aldrich).

### Immunofluorescence staining

Oocyte and testis samples were fixed overnight in 4% PFA in artificial seawater, embedded in OCT compound, serially sectioned on a cryostat and collected onto Superfrost Ultra Plus (Menzel-Gläser). 20 μm-frozen sections were permeabilized in 1 × phosphate-buffered saline (PBS) + 1% Triton X-100 (PBS-T) twice for 5 min at room temperature (RT) and incubated in blocking solution (PBS-T + 10% normal goat serum) for 1 h at RT. Synapsin rabbit polyclonal antibodies (G-304 rabbit E-domain specific) were diluted 1:100 in blocking solution and applied overnight at 4 °C. After three PBS-T washes for 10 min, sections were incubated in Alexa Fluor® 488-conjugated anti-rabbit secondary antibody (1:1000 in blocking solution; Thermo Fisher Scientific), Alexa Fluor™ 647 Phalloidin (Life Technologies, Milan, Italy), and DAPI/Hoechst (1:1000) 2 h at RT. Tissues were rinsed several times and mounted in ProLong Gold antifade reagent (Life Technologies, Milan, Italy). Sections were imaged by confocal microscopy (SP8, Leica Microsystems GmbH, Wetzlar, Germany). *Z*-stack images were acquired in 1-µm steps and three-dimensional reconstructions were generated using Leica Application Suit X software (LAS-X). Offline analysis for immunofluorescence quantification was performed using ImageJ with the Corrected total fluorescence method^53^. Fluorescence intensity in regions of interest was quantified in single section planes (1 µm).

### Molecular modeling

A structural model of the folded *O. vulgaris* synapsin C domain (residues 91 to 391) was obtained using the I-TASSER structure prediction web server^25^. I-TASSER is a hierarchical approach to protein structure prediction that ranked first in recent community-wide experiments (CASP7 to CASP12)^54^. Given a target sequence, it first identifies suitable structure templates in the PDB database based on sequence similarity (called threading), and models *ab initio* the regions for which no template can be found; then, using stochastic simulations, it assembles the template fragments into full-length conformations and refines them. For *O. vulgaris* synapsin C domain, I-TASSER identified rat synapsin II C domain as the best template (PDB code 1i7l). The two domain sequences show 65% identity, and the alignment to predict the structure extended over 95% of the residues.

The two parameters used by I-TASSER to estimate the quality of a predicted model are the C-score and the TM-score. The C-score is calculated based on the significance of the template alignments and the convergence parameters of the structure assembly simulations. The C-score is typically in the range [−5, 2], where the higher the value, the higher the confidence of the model. The TM-score is a measure of the difference between two structures that is more sensitive to global similarities than local differences, whose estimate for a predicted structure with respect to the actual (unknown) fold has been demonstrated to be correlated to the C-score. A predicted TM-score > 0.5 indicates a model of correct topology, while a TM-score < 0.17 corresponds to random similarity.

### Statistical analysis

Statistical analysis was performed using SigmaPlot 13.0 (Systat Software, Inc.). Normality of the datasets was first assessed with the Shapiro-Wilk normality test. Student’s t-test was used to assess the difference between two experimental groups. Three technical replicates were used (n = 3). p values < 0.05 were considered significant (*p < 0.05, **p < 0.01, ***p < 0.001).

## Supplementary information

Additional File 1

Additional file 2

Additional file 3

Additional file 4

Additional file 5

Additional info on probe length of additional file.5a

## Data Availability

All data generated during this study are included in the Supplementary Information files (Additional Files 1–5).
