# Supplementary material for: Synapsins are expressed at neuronal and non-neuronal locations in Octopus vulgaris
Source: Sci Rep. 2019 Oct 28;9:15430. doi: 10.1038/s41598-019-51899-y (PMC6817820; doi:10.1038/s41598-019-51899-y)
Supplement: Supplementary file 1 — Additional File 1 [file 41598_2019_51899_MOESM1_ESM.pdf]

Synapsins are expressed at neuronal and non-neuronal locations in Octopus vulgaris

Federica Maiole[1,2+], Giulia Tedeschi[2,3+], Simona Candiani[4*], Luca Maragliano[1,5], Fabio Benfenati[1,5], Letizia Zullo[1,5*]

Supplementary Info:
Additional File 1: Project for the cloning of the synapsin transcripts from *Octopus vulgaris*.

(**a**) Genomic organization of *Octopus bimaculoides* synapsin gene locus from JGI Metazome. The different synapsin isoforms identified in the present work from *Octopus vulgaris* are shown at the level of specific exons of the corresponding genomic sequence of *O. bimaculoides*. Change in the exon usage driven by alternative splicing generates the Syn-long (**a^{I}**) and the Syn-short (**a^{II}**) isoforms. In particular, the Syn-short isoform lacks the Exon 11 that is present in the Syn-long isoform. The Syn8.2 isoform (**a^{I}**, framed in red) is a variation of the Syn-long isoform and present an addition of 15 nucleotides at the border of exon 5. For clarity, *O. bimaculoides* exon 1 and 5 sequences are reported in full and highlighted in yellow (**a^{III}**). The sequence of the exon 1 of *O. bimaculoides* is characterized by the presence of a stop translation codon upstream isoleucine thus supporting the occurrence of a non-AUG start codon as it was found in *O. vulgaris* synapsin. (**b-c**) To isolate the complete sequence of *O. vulgaris* synapsin, overlapped primers have been designed based on the sequence from *O. bimaculoides*. The position of the primers is indicated on the deduced sequence of *O. vulgaris* Syn-long. The sequence of the primers used for synapsins cloning is also reported in (**c**) (in bold are nucleotides different from *O. bimaculoides* and *O. vulgaris*). (**d**) *O. vulgaris* Syn-long full sequence with the primers used for the cloning highlighted in different colors (same as in (**b**)). (**e**) Electrophoresis gels showing the amplification products by PCR with the following primers: 218-219 (**e^{I}**), 200-201 (**e^{II}**), 192-199 (**e^{III}**), 198-199 (**e^{IV}**). The long and short isoforms are visible in **e^{III}** and **e^{IV}**. In **e^{III}** different cDNAs were used to amplify the products and in lane 2 non-specific PCR product was visible. Both short and long isoforms were identified from all three tissues although a different level of expression of the short isoform can be noticed. (**f-g**) Alignment of the nucleotide sequences (**f**) and of the predicted protein sequences (**g**) of the three synapsin transcripts from *O. vulgaris* obtained by sequencing of overlapping clones. For comparison the sequence of synapsin from *O. bimaculoides* is also shown. Identical residues are highlighted in *yellow*, identical residues in at least 50% of sequences in *light blue* and those conserved in at least 50 % in *green*. (**h**) Chromatogram of DNA sequences from two clones amplified by ovary and brain using primer 218-219 and sequenced from both ends showing a stop translation codon upstream isoleucine (highlighted in yellow). (**i**) Selected chromatogram files with DNA sequences of the short isoform from clones amplified by PCR with 192-199 primers from ovary, testis and brain are reported. In yellow is shown the region where the long isoform has 102 bp more. (**j**) Selected chromatogram files with DNA sequences of the long isoform from clones amplified by PCR with 192-199 primers from ovary, testis and brain showing the region where the long isoform has 102 bp more than the short isoform are reported. In yellow and blue are labelled the regions in common and not between the short and long isoforms, respectively. (**k**) A chromatogram file with DNA sequence of clones 17, 20, 15 and 8 amplified by PCR with 192-193 primers. Clone 15 amplified from ovary contains the Syn8.2 isoform showing a stretch of 15 extra nucleotides encoding for 5 amino acids (typical of Syn8.2 isoform and located at the central part of domain C, highlighted in yellow). (**l**) Full Contig Assembly of clones 17, 20, 15 and 8 amplified by

PCR with 192-193 primers showing the Syn8.2 isoform from clone 15 with the stretch of 15 extra nucleotides encoding for 5 amino acids.

Additional File 1: Project for the cloning of the synapsin transcripts from *Octopus vulgaris*.

(**a**) Genomic organization of *Octopus bimaculoides* synapsin gene locus from JGI Metazome. The different synapsin isoforms identified in the present work from *Octopus vulgaris* are shown at the level of specific exons of the corresponding genomic sequence of *O. bimaculoides*. Change in the exon usage driven by alternative splicing generates the Syn-long (**a**^{I}) and the Syn-short (**a**^{II}) isoforms. In particular, the Syn-short isoform lacks the Exon 11 that is present in the Syn-long isoform. The Syn8.2 isoform (**a**^{I}, framed in red) is a variation of the Syn-long isoform and present an addition of 15 nucleotides at the border of exon 5. For clarity, *O. bimaculoides* exon 1 and 5 sequences are reported in full and highlighted in yellow (**a**^{III}). The sequence of the exon 1 of *O. bimaculoides* is characterized by the presence of a stop translation codon upstream isoleucine thus supporting the occurrence of a non-AUG start codon as it was found in *O. vulgaris* synapsin.

(I)

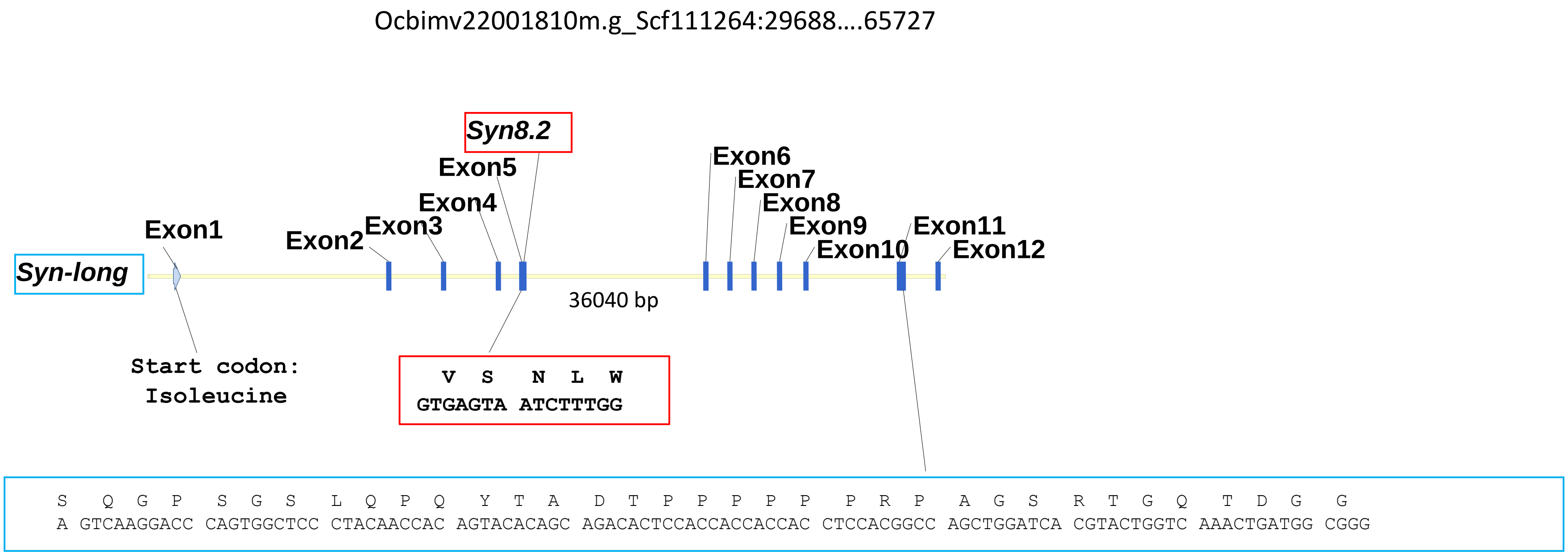

(II)

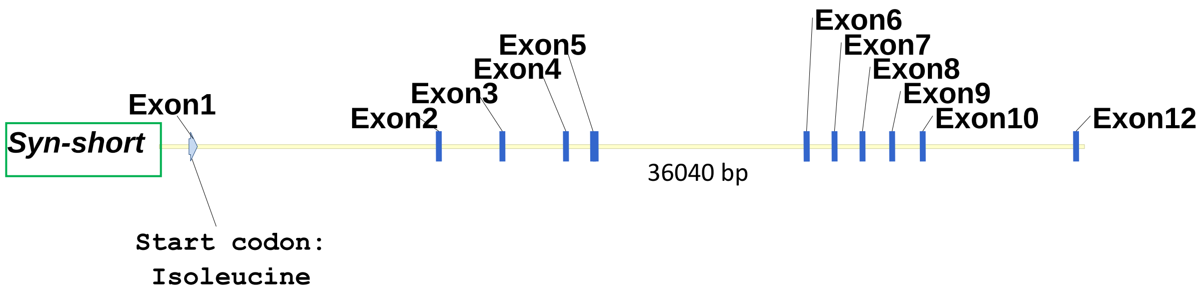

(III)

Exon 1

```
1001                                  ACAAACAATT CTTTCCCCTC TTCCTTCCTT TTTTGTCTCT CTCCAAAAGT CTTTCTCATT TTTTCCCTTA
      · I  V  P   T  K  P  V   K  I  S   F  S  T   F  K  D  N   F  S  T   G  V  S   F  L  R  R   R  F  S   S  G  D
1101  AATCGTTCCA ACAAAACCCG TCAAAATTTC GTTCTCAACA TTCAAGGACA ACTTCTCGAC GGGAGTCAGC TTCCTCCGGA GACGCTTCAG CTCAGGTGAC
       L  Q  G  E   L  R  D   A  Q  E   Q  G  Q  V   P  V  L   P  I  R   K  G  P  S   P  S  A   P  S  S   P  S  K  T ·
1201  CTCCAGGGCG AGCTACGTGA TGCCCAAGAG CAAGGCCAGG TCCCCGTGCT TCCGATACGG AAGGGGCCTT CGCCGAGTGC GCCCAGTTCC CCATCGAAGA
      ·  S  A  V   G  I  A   K  G  I  F   S  G  Q   R  S  N   V  N  K  D   R  C  K   T  L  L   V  I  D  D   P  H  T ·
1301  CCAGCGCGGT CGGGATTGCC AAGGGCATAT TCTCAGGTCA GAGGTCAAAT GTCAACAAGG ACCGATGTAA GACCCTCCTT GTCATTGACG ATCCCCACAC
      · D
1401  CGACTGGTAA GGCCCCTATT TTTGTTCCTT GGGATTATTT TA
```

Exon 5

```
16701   ACACCATCTA GCAATCCATA AAACTCCCTC CCTCTCTCTC ACTTGTACAC CATCACTCTT TCTCTCCCAC ATCCTTCTCC CCTCTCTCTG TATTTCCCTC
                                                                      M  A  H   L  I  Q   I  Q  K   K  L  G  P ·
16801   CTTCTCCTCC TCCCTCCCTC TCTCTCTCTC TCTTTCTCTC TCACTGCCCC TCTCTCCCCA CAGATGGCAC ACTTAATCCA GATCCAAAAG AAACTTGGAC
        · D  K  F   P  L  I   D  Q  A  Y   Y  P  N   H  K  E   M  V  S  N   L  W
16901   CGGACAAGTT CCCATTGATC GACCAAGCGT ATTATCCAAA CCACAAGGAA ATGGTGAGTA ATCTTTGGGT GAGTTATTCT TGATCCTTTG CCTGTACCCT
17001   TTCATTCGCT TTGGAAACTG TACTTCTGTT AGTTCATTGT TAGACATCTA TAGACTAAAC GTTGCAGAGA GCATCCAAAA TTTAGAGGTT GTAGGTCAAG
17101   ATGCCCCCTT CCCTCACACA CACACATTCA GATAACGTCA AACCATGCCC CACTACTTTA AAAAAACGTT GGTTCAGATA
```

(**b-c**) To isolate the complete sequence of *O. vulgaris* synapsin, overlapped primers have been designed based on the sequence from *O. bimaculoides*. The position of the primers is indicated on the deduced sequence of *O. vulgaris* Syn-long. The sequence of the primers used for synapsins cloning is also reported in (**c**) (in bold are nucleotides different from *O. bimaculoides* and *O. vulgaris*).

b)

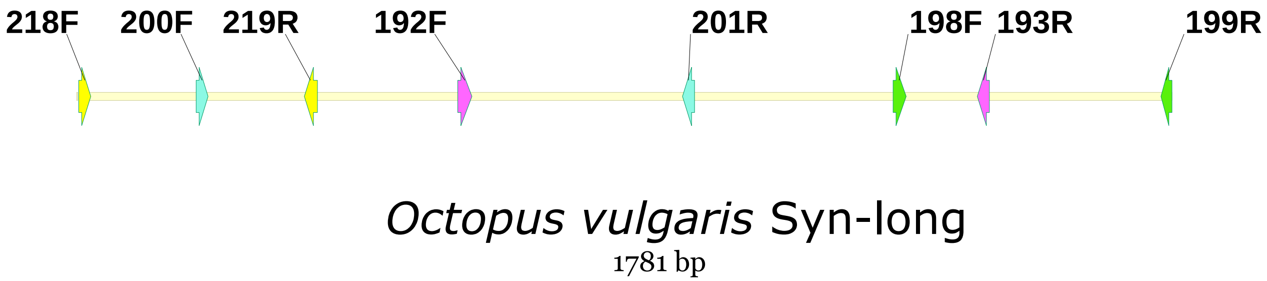

c)

Name	Sequence (5’-3’)
192 (F)	**A**GGAATGGCACCAGAGTTGTCAG
193 (R)	GGGAGGTCCGCCATCAGTTTG
198 (F)	TGAGATGGTGCTGCAGAAAA
199 (R)	CGCCTAACAATCCCCAGAC
200 (F)	CCAACAAAACCCGTCAAAAT
201 (R)	TTGGTCACAGCCAC**A**ACACT
218 (F)	TCTTCAGGGGCCTTCTACAA
219 (R)	CTGGTCTTCGATGGGGAACT

(**d**) *O. vulgaris* Syn-long full sequence with the primers used for the cloning highlighted in different colors (same as in (**b**)).

```
      F  R  G   L  L  Q   S  S  F  N   F  F  *   K  R  S   S  F  H  F   F  P  F   S  V  C   L  S  H  I   *  Q  K ·
  1  TCTTCAGGGG CCTTCTACAA TCCTCCTTCA ATTTTTTTTA AAAAAGGTCA TCCTTCCATT TCTTTCCTTT CTCTGTCTGT CTGTCTCATA TTTAACAAAA
     · E  K  Q   Q  N  K  Q   F  F  P   L  F  L   P  F  F  S   L  S  K   S  L  S   H  F  F  P   *  I  V   P  T  K
101  AGAAAAACAA CAAAACAAAC AATTCTTTCC CCTCTTCCTT CCTTTTTTTT CTCTCTCCAA AAGTCTTTCT CATTTTTTCC CTTAAATCGT TCCAACAAAA
      P  V  K  I   S  F  S   T  F  K   D  N  F  S   T  G  V   G  F  L   R  R  R  F   S  S  G   D  L  Q   G  E  L  R ·
201  CCCGTCAAAA TTTCGTTCTC AACATTCAAG GACAACTTCT CGACGGGAGT CGGCTTCCTC CGGAGACGCT TCAGCTCAGG TGACCTCCAG GGCGAGCTAC
     ·  D  A  Q   E  Q  G   Q  V  P  V   L  P  I   R  K  G   P  S  P  S   A  P  S   S  P  S   K  T  S  A   V  G  I ·
301  GTGATGCCCA AGAGCAAGGC CAGGTCCCCG TGCTTCCGAT ACGGAAGGGG CCTTCGCCGA GTGCGCCCAG TTCCCCATCG AAGACCAGCG CGGTCGGGAT
     · A  K  G   I  F  S  G   Q  R  S   N  V  N   K  D  R  C   K  T  L   L  V  I   D  D  P  H   T  D  W   S  K  Y
401  TGCCAAGGGC ATATTCTCAG GTCAGAGGTC AAATGTCAAC AAGGACCGAT GTAAGACCCT CCTTGTCATT GACGATCCCC ACACCGACTG GTCGAAATAT
      F  R  G  K   K  L  F   G  D  W   D  I  R  I   E  Q  A   E  F  P   E  I  N  V   A  A  Y   T  D  Q   G  T  M  V ·
```

```
501   TTCCGCGGTA AGAAGCTATT TGGTGACTGG GACATACGCA TAGAGCAGGC AGAGTTTCCT GAAATCAATG TTGCTGCCTA CACAGACCAA GGGACCATGG
       · D  I  Q   V  L  R   N  G  T  R   V  V  R   S  F  K   P  D  F  V   L  V  R   Q  H  V   R  D  A  C   E  D  W ·
601   TCGACATTCA AGTATTGCGG AATGGCACCA GAGTTGTCAG GTCTTTCAAG CCAGACTTTG TGCTCGTCAG ACAACATGTG CGTGACGCTT GTGAGGACTG
       ·R  N  L   I  M  G  F   H  Y  G   G  V  P   S  I  N  S   M  D  S   I  Y  N   F  Q  D  K   P  W  V   M  A  H
701   GCGTAACTTG ATCATGGGGT TCCATTATGG CGGTGTTCCT AGTATTAATT CTATGGATTC CATTTACAAC TTTCAAGATA AACCTTGGGT GATGGCACAC
        L  I  Q  I   Q  K  K   L  G  P   D  K  F  P   L  I  D   Q  A  Y   Y  P  N  H   K  E  M   L  V  T   P  K  F  P ·
801   TTAATCCAGA TCCAAAAGAA ACTTGGACCG GACAAGTTCC CATTGATCGA CCAAGCGTAT TATCCAAACC ACAAGGAAAT GCTGGTCACT CCAAAATTTC
       · V  V  V   K  I  G   H  A  H  S   G  M  G   K  V  K   I  D  H  H   H  A  F   Q  D  I   S  S  V  V   A  V  T ·
901   CAGTTGTGGT GAAGATTGGA CATGCTCATT CTGGTATGGG AAAGGTGAAG ATCGACCACC ATCATGCCTT CCAAGACATC TCCAGTGTCG TGGCTGTGAC
       ·K  T  Y   A  T  T  E   P  F  I   D  C  K   Y  D  I  R   V  Q  K   I  G  N   N  Y  K  A   F  I  R   K  S  I
1001  CAAGACGTAT GCCACCACAG AGCCATTCAT TGACTGCAAG TACGACATCC GTGTGCAGAA GATAGGCAAC AATTACAAGG CATTCATTCG CAAATCCATA
        S  G  N  W   K  A  N   T  G  S   A  M  L  E   Q  I  A   M  N  D   R  Y  K  L   W  V  D   E  C  S   Q  M  F  G ·
1101  TCAGGAAACT GGAAAGCCAA CACCGGGTCA GCAATGCTGG AACAGATAGC TATGAATGAC AGATATAAGC TGTGGGTTGA TGAGTGCAGC CAAATGTTTG
       · G  L  D   I  V  A   V  E  A  L   Q  G  K   D  G  R   E  Y  V  I   E  V  N   D  S  S   M  V  L  L   G  E  T ·
1201  GAGGTCTGGA TATTGTAGCT GTGGAAGCCC TGCAGGGTAA AGATGGCCGT GAATATGTCA TTGAGGTGAA CGACTCATCA ATGGTTCTGT TAGGGGAGAC
       ·Q  E  E   D  R  R  L   I  S  E   M  V  L   Q  K  M  Q   I  Y  C   K  P  G   M  S  Q  G   P  S  G   S  L  Q
1301  TCAAGAGGAG GACCGACGCC TCATCTCTGA GATGGTGCTG CAGAAAATGC AGATTTACTG CAAACCAGGA ATGAGTCAAG GACCCAGTGG CTCCCTACAA
        Q  Q  Y  T   A  D  T   P  P  P   P  P  P  R   P  A  G   S  R  T   G  Q  T  D   G  G  P   P  G  Q   Q  P  G  V ·
1401  CAACAGTACA CGGCAGACAC TCCACCACCA CCACCTCCAC GGCCAGCTGG ATCACGTACT GGTCAAACTG ATGGCGGACC TCCCGGTCAG CAACCTGGTG
       · P  A  R   P  G  Q   P  G  Q  G   P  P  Q   A  A  G   Q  P  P  P   P  P  P   Q  Q  Q   Q  Q  Q  Q   Q  H  Q ·
1501  TTCCAGCAAG GCCCGGACAG CCAGGACAGG GTCCACCGCA AGCGGCGGGG CAGCCGCCGC CACCACCACC GCAGCAGCAA CAACAACAAC AACAACACCA
       ·Q  Q  Q   S  Q  Q  Q   H  P  H   Q  Q  Q   Q  S  G  N   Q  A  H   P  P  A   P  G  G  Q   G  Q  D   E  D  T
1601  GCAACAACAA AGCCAACAGC AGCACCCTCA CCAACAACAG CAGTCAGGGA ACCAGGCGCA CCCGCCGGCC CCAGGGGGGC AGGGCCAAGA TGAGGACACA
        M  Q  N  L   R  K  T   F  A  G   I  F  G  D   M  *  S   K  F  *   G  S  G  D   C  *  A
1701  ATGCAAAACC TTCGGAAGAC TTTCGCTGGA ATTTTCGGGG ACATGTGAAG CAAATTTTAA GGGTCTGGGG ATTGTTAGGC G
```

($\mathbf{e}$) Electrophoresis gels showing the amplification products by PCR with the following primers: 218-219 ($\mathbf{e}^{I}$), 200-201 ($\mathbf{e}^{II}$), 192-199 ($\mathbf{e}^{III}$), 198-199 ($\mathbf{e}^{IV}$). The long and short isoforms are visible in $\mathbf{e}^{III}$ and $\mathbf{e}^{IV}$. In $\mathbf{e}^{III}$ different cDNAs were used to amplify the products and in lane 2 non-specific PCR product was visible. Both short and long isoforms were identified from all three tissues although a different level of expression of the short isoform can be noticed.

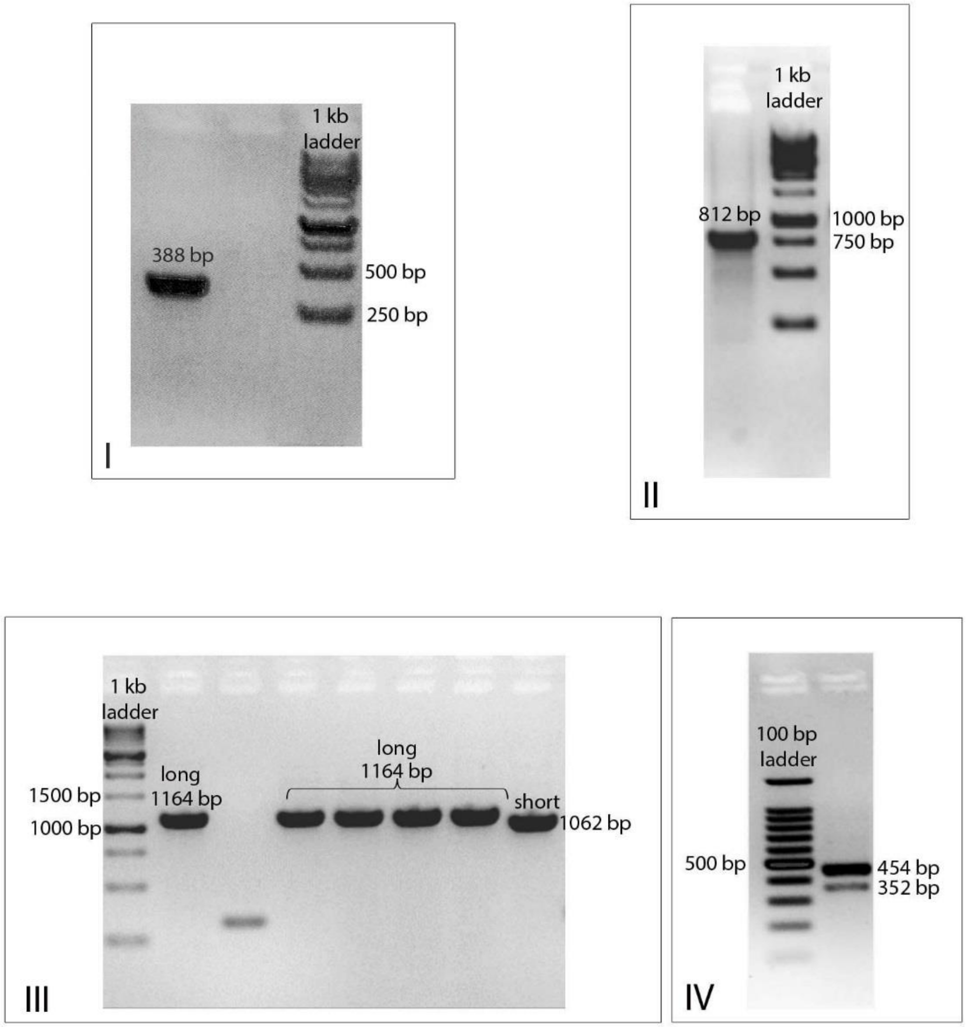

(**f-g**) Alignment of the nucleotide sequences (**f**) and of the predicted protein sequences (**g**) of the three synapsin transcripts from *O. vulgaris* obtained by sequencing of overlapping clones. For comparison the sequence of synapsin from *O. bimaculoides* is also shown. Identical residues are highlighted in *yellow*, identical residues in at least 50% of sequences in *light blue* and those conserved in at least 50 % in *green*.

f)

```
                                 1                                                                              80
Obimaculoides_Synlong      (1)   TCTTCAGGGGCCTTCTACAATCTTCCTTCAATTTTTTTTAAAAAAAGGTCATCCTCCCATTTCTTTCTTTTCTCTGTCTG
    Ovulgaris_Synlong      (1)   TCTTCAGGGGCCTTCTACAATCCTCCTTCAATTTTTTTTAAAAAA-GGTCATCCTTCCATTTCTTTCCTTTCTCTGTCTG
   Ovulgaris_Synshort      (1)   --------------------------------------------------------------------------------
     Ovulgaris_Syn8.2      (1)   --------------------------------------------------------------------------------
                                 81                                                                            160
Obimaculoides_Synlong     (81)   TCTTTCTCATATTTAACAAAAAAAGGAAAACAACAAAACAAACAATTCTTTCCCCTCTTCCTTCCTTTTTTGTCTCTCTC
    Ovulgaris_Synlong     (80)   TCTGTCTCATATTTAACAAAAAGA--AAAACAACAAAACAAACAATTCTTTCCCCTCTTCCTTCCTTTTTTTTCTCTCTC
   Ovulgaris_Synshort      (1)   --------------------------------------------------------------------------------
     Ovulgaris_Syn8.2      (1)   --------------------------------------------------------------------------------
                                 161                                                                           240
Obimaculoides_Synlong    (161)   CAAAAGTCTTTCTCATTTTTTCCCTTAAATCGTTCCAACAAAACCCGTCAAAATTTCGTTCTCAACATTCAGGACAACT
    Ovulgaris_Synlong    (158)   CAAAAGTCTTTCTCATTTTTTCCCTTAAATCGTTCCAACAAAACCCGTCAAAATTTCGTTCTCAACATTCAGGACAACT
   Ovulgaris_Synshort      (1)   ----------------------------ATCGTTCCAACAAAACCCGTCAAAATTTCGTTCTCAACATTCAGGACAACT
     Ovulgaris_Syn8.2      (1)   ----------------------------ATCGTTCCAACAAAACCCGTCAAAATTTCGTTCTCAACATTCAGGACAACT
                                 241                                                                           320
Obimaculoides_Synlong    (241)   TCTCGACGGGAGTCAGCTTCCTCCGGAGACGCTTCAGCTCAGGTGACCTCCAGGGCGAGCTACGTGATGCCCAAGAGCAA
    Ovulgaris_Synlong    (238)   TCTCGACGGGAGTCGGCTTCCTCCGGAGACGCTTCAGCTCAGGTGACCTCCAGGGCGAGCTACGTGATGCCCAAGAGCAA
   Ovulgaris_Synshort     (53)   TCTCGACGGGAGTCGGCTTCCTCCGGAGACGCTTCAGCTCAGGTGACCTCCAGGGCGAGCTACGTGATGCCCAAGAGCAA
     Ovulgaris_Syn8.2     (53)   TCTCGACGGGAGTCAGCTTCCTCCGGAGACGCTTCAGCTCAGGTGACCTCCAGGGCGAGCTACGTGATGCCCAAGAGCAA
```

```
                                321                                                                            400
Obimaculoides_Synlong    (321)  GGCCAGGTCCCCGTGCTTCCGATACGGAAGGGGCCTTCGCCGAGTGCGCCCAGTTCCCCATCGAAGACCAGCGCGGTCGG
     Ovulgaris_Synlong   (318)  GGCCAGGTCCCCGTGCTTCCGATACGGAAGGGGCCTTCGCCGAGTGCGCCCAGTTCCCCATCGAAGACCAGCGCGGTCGG
    Ovulgaris_Synshort   (133)  GGCCAGGTCCCCGTGCTTCCGATACGGAAGGGGCCTTCGCCGAGTGCGCCCAGTTCCCCATCGAAGACCAGCGCGGTCGG
      Ovulgaris_Syn8.2   (133)  GGCCAGGTCCCCGTGCTTCCGATACGGAAGGGGCCTTCGCCGAGTGCGCCCAGTTCCCCATCGAAGACCAGCGCGGTCGG
                                401                                                                            480
Obimaculoides_Synlong    (401)  GATTGCCAAGGGCATATTCTCAGGTCAGAGGTCAAATGTCAACAAGGACCGATGTAAGACCCTCCTTGTCATTGACGATC
     Ovulgaris_Synlong   (398)  GATTGCCAAGGGCATATTCTCAGGTCAGAGGTCAAATGTCAACAAGGACCGATGTAAGACCCTCCTTGTCATTGACGATC
    Ovulgaris_Synshort   (213)  GATTGCCAAGGGCATATTCTCAGGTCAGAGGTCAAATGTCAACAAGGACCGATGTAAGACCCTCCTTGTCATTGACGATC
      Ovulgaris_Syn8.2   (213)  GATTGCCAAGGGCATATTCTCAGGTCAGAGGTCAAATGTCAACAAGGACCGATGTAAGACCCTCCTTGTCATTGACGATC
                                481                                                                            560
Obimaculoides_Synlong    (481)  CCCACACCGACTGGTCGAAATATTTCCGCGGTAAGAAGCTATTTGGTGACTGGGACATACGAATAGAGCAGGCAGAGTTT
     Ovulgaris_Synlong   (478)  CCCACACCGACTGGTCGAAATATTTCCGCGGTAAGAAGCTATTTGGTGACTGGGACATACGCATAGAGCAGGCAGAGTTT
    Ovulgaris_Synshort   (293)  CCCACACCGACTGGTCGAAATATTTCCGCGGTAAGAAGCTATTTGGTGACTGGGACATACGCATAGAGCAGGCAGAGTTT
      Ovulgaris_Syn8.2   (293)  CCCACACCGACTGGTCGAAATATTTCCGCGGTAAGAAGCTATTTGGTGACTGGGACATACGCATAGAGCAGGCAGAGTTT
                                561                                                                            640
Obimaculoides_Synlong    (561)  CCTGAAATCAATGTTGCTGCCTACACAGACCAAGGGACCATGGTCGACATTCAAGTATTGAGGAATGGCACCAGAGTTGT
     Ovulgaris_Synlong   (558)  CCTGAAATCAATGTTGCTGCCTACACAGACCAAGGGACCATGGTCGACATTCAAGTATTGCGGAATGGCACCAGAGTTGT
    Ovulgaris_Synshort   (373)  CCTGAAATCAATGTTGCTGCCTACACAGACCAAGGGACCATGGTCGACATTCAAGTATTGCGGAATGGCACCAGAGTTGT
      Ovulgaris_Syn8.2   (373)  CCTGAAATCAATGTTGCTGCCTACACAGACCAAGGGACCATGGTCGACATTCAAGTATTGCGGAATGGCACCAGAGTTGT
                                641                                                                            720
Obimaculoides_Synlong    (641)  CAGGTCTTTCAAGCCAGACTTTGTGCTCGTCAGACAACATGTGCGTGACGCTTGTGAGGACTGGCGTAACCTGATCATGG
     Ovulgaris_Synlong   (638)  CAGGTCTTTCAAGCCAGACTTTGTGCTCGTCAGACAACATGTGCGTGACGCTTGTGAGGACTGGCGTAACTTGATCATGG
    Ovulgaris_Synshort   (453)  CAGGTCTTTCAAGCCAGACTTTGTGCTCGTCAGACAACATGTGCGTGACGCTTGTGAGGACTGGCGTAACTTGACCATGG
      Ovulgaris_Syn8.2   (453)  CAGGTCTTTCAAGCCAGACTTTGTGCTCGTCAGACAACATGTGCGTGACGCTTGTGAGGACTGGCGTAACTTGATCATGG
                                721                                                                            800
Obimaculoides_Synlong    (721)  GGTTCCATTATGGCGGTGTTACTAGTATTAATTCTATGGATTCCATTTACAACTTTCAAGATAAACCTTGGGTGATGGCA
     Ovulgaris_Synlong   (718)  GGTTCCATTATGGCGGTGTTCCTAGTATTAATTCTATGGATTCCATTTACAACTTTCAAGATAAACCTTGGGTGATGGCA
    Ovulgaris_Synshort   (533)  GGTTCCATTATGGCGGTGTTCCTAGTATTAATTCTATGGATTCCATTTACAACTTTCAAGATAAACCTTGGGTGATGGCA
      Ovulgaris_Syn8.2   (533)  GGTTCCATTATGGCGGTGTTCCTAGTATTAATTCTATGGATTCCATTTACAACTTTCAAGATAAACCTTGGGTGATGGCA
```

```
                                801                                                                            880
Obimaculoides_Synlong    (801) CACTTAATCCAGATCCAAAAGAAACTTGGACCGGACAAGTTCCCATTGATCGACCAAGCGTATTATCCAAACCACAAGGA
     Ovulgaris_Synlong   (798) CACTTAATCCAGATCCAAAAGAAACTTGGACCGGACAAGTTCCCATTGATCGACCAAGCGTATTATCCAAACCACAAGGA
   Ovulgaris_Synshort    (613) CACTTAATCCAGATCCAAAAGAAACTTGGACCGGACAAGTTCCCATTGATCGACCAAGCGTATTATCCAAACCACAAGGA
      Ovulgaris_Syn8.2   (613) CACTTAATCCAGATCCAAAAGAAACTTGGACCGGACAAGTTCCCATTGATCGACCAAGCGTATTATCCAAACCACAAGGA
                                881                                                                            960
Obimaculoides_Synlong    (881) AATG---------------CTGGTCACTCCAAAATTTCCAGTTGTGGTGAAGATTGGACATGCTCATTCTGGTATGGGAA
     Ovulgaris_Synlong   (878) AATG---------------CTGGTCACTCCAAAATTTCCAGTTGTGGTGAAGATTGGACATGCTCATTCTGGTATGGGAA
   Ovulgaris_Synshort    (693) AATG---------------CTGGTCACTCCAAAATTTCCAGTTGTGGTGAAGATTGGACATGCTCATTCTGGTATGGGAA
      Ovulgaris_Syn8.2   (693) AATGGTGAGTAATCTTTGGCTGGTCACTCCAAAATTTCCAGTTGTGGTGAAGATTGGACATGCTCATTCTGGTATGGGAA
                                961                                                                           1040
Obimaculoides_Synlong    (946) AGGTGAAGATCGACCACCATCATGCCTTCCAAGACATCTCCAGTGTTGTGGCTGTGACCAAGACATATGCCACCACAGAG
     Ovulgaris_Synlong   (943) AGGTGAAGATCGACCACCATCATGCCTTCCAAGACATCTCCAGTGTCGTGGCTGTGACCAAGACGTATGCCACCACAGAG
   Ovulgaris_Synshort    (758) AGGTGAAGATCGACCACCATCATGCCTTCCAAGACATCTCCAGTGTCGTGGCTGTGACCAAGACGTATGCCACCACAGAG
      Ovulgaris_Syn8.2   (773) AGGTGAAGATCGACCACCATCATGCCTTCCAAGACATCTCCAGTGTCGTGGCTGTGACCAAGACGTATGCCACCACAGAG
                                1041                                                                          1120
Obimaculoides_Synlong   (1026) CCATTCATTGACTGCAAGTACGACATCCGTGTGCAGAAGATAGGCAACAATTACAAGGCATTCATTCGCAAATCCATATC
     Ovulgaris_Synlong  (1023) CCATTCATTGACTGCAAGTACGACATCCGTGTGCAGAAGATAGGCAACAATTACAAGGCATTCATTCGCAAATCCATATC
   Ovulgaris_Synshort    (838) CCATTCATTGACTGCAAGTACGACATCCGTGTGCAGAAGATAGGCAACAATTACAAGGCATTCATTCGCAAATCCATATC
      Ovulgaris_Syn8.2   (853) CCATTCATTGACTGCAAGTACGACATCCGTGTGCAGAAGATAGGCAACAATTACAAGGCATTCATTCGCAAATCCATATC
                                1121                                                                          1200
Obimaculoides_Synlong   (1106) TGGAAACTGGAAAGCCAATACCGGGTCAGCAATGCTGGAACAGATAGCTATGAATGACAGATATAAGCTGTGGGTTGATG
     Ovulgaris_Synlong  (1103) AGGAAACTGGAAAGCCAACACCGGGTCAGCAATGCTGGAACAGATAGCTATGAATGACAGATATAAGCTGTGGGTTGATG
   Ovulgaris_Synshort    (918) AGGAAACTGGAAAGCCAACACCGGGTCAGCAATGCTGGAACAGATAGCTATGAATGACAGATATAAGCTGTGGGTTGATG
      Ovulgaris_Syn8.2   (933) AGGAAACTGGAAAGCCAACACCGGGTCAGCAATGCTGGAACAGATAGCTATGAATGACAGATATAAGCTGTGGGTTGATG
                                1201                                                                          1280
Obimaculoides_Synlong   (1186) AGTGCAGCCAAATGTTTGGAGGTCTGGATATTGTAGCTGTGGAAGCCCTGCAGGGTAAAGACGGCCGTGAATATATCATT
     Ovulgaris_Synlong  (1183) AGTGCAGCCAAATGTTTGGAGGTCTGGATATTGTAGCTGTGGAAGCCCTGCAGGGTAAAGATGGCCGTGAATATGTCATT
   Ovulgaris_Synshort    (998) AGTGCAGCCAAATGTTTGGAGGTCTGGATATTGTAGCTGTGGAAGCCCTGCAGGGTAAAGATGGCCGTGAATATGTCATT
      Ovulgaris_Syn8.2  (1013) AGTGCAGCCAAATGTTTGGAGGTCTGGATATTGTAGCTGTGGAAGCCCTGCAGGGTAAAGATGGCCGTGAATATGTCATT
```

```
                                1281                                                                            1360
Obimaculoides_Synlong   (1266)  GAGGTGAACGACTCATCAATGGTTCTGTTAGGAGAGACTCAAGAGGAGGACCGACGCCTCATCTCTGAGATGGTGCTGCA
   Ovulgaris_Synlong    (1263)  GAGGTGAACGACTCATCAATGGTTCTGTTAGGGGAGACTCAAGAGGAGGACCGACGCCTCATCTCTGAGATGGTGCTGCA
  Ovulgaris_Synshort    (1078)  GAGGTGAACGACTCATCAATGGTTCTGTTAGGGGAGACTCAAGAGGAGGACCGACGCCTCATCTCTGAGATGGTGCTGCA
    Ovulgaris_Syn8.2    (1093)  GAGGTGAACGACTCATCAATGGTTCTGTTAGGGGAGACTCAAGAGGAGGACCGACGCCTCATCTCTGAGATGGTGCTGCA
                                1361                                                                            1440
Obimaculoides_Synlong   (1346)  GAAAATGCAGATTTACTGCAAACCAGGAATGAATCAAGGACCCAGTGGCTCCCTACAACCACAGTACACAGCAGACACTC
   Ovulgaris_Synlong    (1343)  GAAAATGCAGATTTACTGCAAACCAGGAATGAGTCAAGGACCCAGTGGCTCCCTACAACAACAGTACACGGCAGACACTC
  Ovulgaris_Synshort    (1158)  GAAAATGCAGATTTACTGCAAACCAGGAATGAG-----------------------------------------------
    Ovulgaris_Syn8.2    (1173)  GAAAATGCAGATTTACTGCAAACCAGGAATGAGTCAAGGACCCAGTGGCTCCCTACAACAACAGTACACGGCAGACACTC
                                1441                                                                            1520
Obimaculoides_Synlong   (1426)  CACCACCACCACCTCCACGGCCAGCTGGATCACGTACTGGTCAAACTGATGGCGGACCTCCCGGTCAGCAACCAGGTGTC
   Ovulgaris_Synlong    (1423)  CACCACCACCACCTCCACGGCCAGCTGGATCACGTACTGGTCAAACTGATGGCGGACCTCCCGGTCAGCAACCTGGTGTT
  Ovulgaris_Synshort    (1191)  -------------------------------------------------------ACCTCCCGGTCAGCAACCTGGTGTT
    Ovulgaris_Syn8.2    (1253)  CACCACCACCACCTCCACGGCCAGCTGGATCACGTACTGGTCAAACTGATGGCGGACCTCCCGGTCAGCAACCTGGTGTC
                                1521                                                                            1600
Obimaculoides_Synlong   (1506)  CCAGCAAGGCCCGGACAGCCAGGACAGGGTCCACCGCAAGCAGCGGGGCAGCCACCGCCACCACCACCGCAGCAGCAACA
   Ovulgaris_Synlong    (1503)  CCAGCAAGGCCCGGACAGCCAGGACAGGGTCCACCGCAAGCGGCGGGGCAGCCGCCGCCACCACCACCGCAGCAGCAACA
  Ovulgaris_Synshort    (1216)  CCAGCAAGGCCCGGACAGCCAGGACAGGGTCCACCGCAAGCGGCGGGGCAGCCGCCGCCACCACCACCGCAGCAGCAACA
    Ovulgaris_Syn8.2    (1333)  CCAGCAAGGCCCGGACAGCCAGGACAGGGTCCACCGCAAGCGGCGGGGCAGCCGCCGCCACCACCACCGCAGCAGCAACA
                                1601                                                                            1680
Obimaculoides_Synlong   (1586)  ACAACAACAACAACACCAGCAGCAACAACAAAGCCAACAGCAGCACCCTCACCAACAACAGCAGTCAGGGAACCAGGCGC
   Ovulgaris_Synlong    (1583)  ACAACAACAACAACACCAGCA---ACAACAAAGCCAACAGCAGCACCCTCACCAACAACAGCAGTCAGGGAACCAGGCGC
  Ovulgaris_Synshort    (1296)  ACAACAACAACAACACCAGCA---ACAACAAAGCCAACAGCAGCACCCTCACCAACAACAGCAGTCAGGGAACCAGGCGC
    Ovulgaris_Syn8.2    (1413)  ACAACAACAACAACACCAGCA---ACAACAAAGCCAACAGCAGCACCCTCACCAACAACAGCAGTCAGGGAACCAGGCGC
                                1681                                                                            1760
Obimaculoides_Synlong   (1666)  ACCCGCCGGCCCCAGGGGGGCAGGGCCAAGATGAGGACACAATGCAAAACCTTCGGAAGACTTTCGCTGGAATTTTCGGG
   Ovulgaris_Synlong    (1660)  ACCCGCCGGCCCCAGGGGGGCAGGGCCAAGATGAGGACACAATGCAAAACCTTCGGAAGACTTTCGCTGGAATTTTCGGG
  Ovulgaris_Synshort    (1373)  ACCCGCCGGCCCCAGGGGGGCAGGGCCAAGATGAGGACACAATGCAAAACCTTCGGAAGACTTTCGCTGGAATTTTCGGG
    Ovulgaris_Syn8.2    (1490)  ACCCGCCGGCCCCAGGGGGGCAGGGCCAAGATGAGGACACAATGCAAAACCTTCGGAAGACTTTCGCCGGAATTTTCGGG
```

```
                              1761                 1782
Obimaculoides_Synlong  (1746) GACATGTGAAGCAAATTTTAA
    Ovulgaris_Synlong  (1740) GACATGTGAAGCAAATTTTAA
   Ovulgaris_Synshort  (1453) GACATGTGAAGCAAATTTTAA
     Ovulgaris_Syn8.2  (1570) GACATGTGAAGCAAATTTTAA
```

g)

```
                           1                                                                              80
 Obimaculoides_Syn    (1) IVPTKPVKISFSTFKDNFSTGVSFLRRRFSSGDLQGELRDAQEQGQVPVLPIRKGPSPSAPSSPSKTSAVGIAKGIFSGQ
 Ovulgaris_Synlong    (1) IVPTKPVKISFSTFKDNFSTGVGFLRRRFSSGDLQGELRDAQEQGQVPVLPIRKGPSPSAPSSPSKTSAVGIAKGIFSGQ
Ovulgaris_Synshort    (1) IVPTKPVKISFSTFKDNFSTGVGFLRRRFSSGDLQGELRDAQEQGQVPVLPIRKGPSPSAPSSPSKTSAVGIAKGIFSGQ
  Ovulgaris_Syn8.2    (1) IVPTKPVKISFSTFKDNFSTGVSFLRRRFSSGDLQGELRDAQEQGQVPVLPIRKGPSPSAPSSPSKTSAVGIAKGIFSGQ

                          81                                                                             160
 Obimaculoides_Syn   (81) RSNVNKDRCKTLLVIDDPHTDWSKYFRGKKLFGDWDIRIEQAEFPEINVAAYTDQGTMVDIQVLRNGTRVVRSFKPDFVL
 Ovulgaris_Synlong   (81) RSNVNKDRCKTLLVIDDPHTDWSKYFRGKKLFGDWDIRIEQAEFPEINVAAYTDQGTMVDIQVLRNGTRVVRSFKPDFVL
Ovulgaris_Synshort   (81) RSNVNKDRCKTLLVIDDPHTDWSKYFRGKKLFGDWDIRIEQAEFPEINVAAYTDQGTMVDIQVLRNGTRVVRSFKPDFVL
  Ovulgaris_Syn8.2   (81) RSNVNKDRCKTLLVIDDPHTDWSKYFRGKKLFGDWDIRIEQAEFPEINVAAYTDQGTMVDIQVLRNGTRVVRSFKPDFVL

                          161                                                                            240
 Obimaculoides_Syn  (161) VRQHVRDACEDWRNLIMGFHYGGVTSINSMDSIYNFQDKPWVMAHLIQIQKKLGPDKFPLIDQAYYPNHKEML-----VT
 Ovulgaris_Synlong  (161) VRQHVRDACEDWRNLIMGFHYGGVPSINSMDSIYNFQDKPWVMAHLIQIQKKLGPDKFPLIDQAYYPNHKEML-----VT
Ovulgaris_Synshort  (161) VRQHVRDACEDWRNLTMGFHYGGVPSINSMDSIYNFQDKPWVMAHLIQIQKKLGPDKFPLIDQAYYPNHKEML-----VT
  Ovulgaris_Syn8.2  (161) VRQHVRDACEDWRNLIMGFHYGGVPSINSMDSIYNFQDKPWVMAHLIQIQKKLGPDKFPLIDQAYYPNHKEMVSNLWLVT

                          241                                                                            320
 Obimaculoides_Syn  (236) PKFPVVVKIGHAHSGMGKVKIDHHHAFQDISSVVAVTKTYATTEPFIDCKYDIRVQKIGNNYKAFIRKSISGNWKANTGS
 Ovulgaris_Synlong  (236) PKFPVVVKIGHAHSGMGKVKIDHHHAFQDISSVVAVTKTYATTEPFIDCKYDIRVQKIGNNYKAFIRKSISGNWKANTGS
```

```
Ovulgaris_Synshort  (236) PKFPVVVKIGHAHSGMGKVKIDHHHAFQDISSVVAVTKTYATTEPFIDCKYDIRVQKIGNNYKAFIRKSISGNWKANTGS
  Ovulgaris_Syn8.2  (241) PKFPVVVKIGHAHSGMGKVKIDHHHAFQDISSVVAVTKTYATTEPFIDCKYDIRVQKIGNNYKAFIRKSISGNWKANTGS

                          321                                                                          400
 Obimaculoides_Syn  (316) AMLEQIAMNDRYKLWVDECSQMFGGLDIVAVEALQGKDGREYIIEVNDSSMVLLGETQEEDRRLISEMVLQKMQIYCKPG
 Ovulgaris_Synlong  (316) AMLEQIAMNDRYKLWVDECSQMFGGLDIVAVEALQGKDGREYVIEVNDSSMVLLGETQEEDRRLISEMVLQKMQIYCKPG
Ovulgaris_Synshort  (316) AMLEQIAMNDRYKLWVDECSQMFGGLDIVAVEALQGKDGREYVIEVNDSSMVLLGETQEEDRRLISEMVLQKMQIYCKPG
  Ovulgaris_Syn8.2  (321) AMLEQIAMNDRYKLWVDECSQMFGGLDIVAVEALQGKDGREYVIEVNDSSMVLLGETQEEDRRLISEMVLQKMQIYCKPG

                          401                                                                          480
 Obimaculoides_Syn  (396) MNQGPSGSLQPQYTADTPPPPPPRPAGSRTGQTDGGPPGQQPGVPARPGQPGQGPPQAAGQPPPPPPQQQQQQQQHQQQQ
 Ovulgaris_Synlong  (396) MSQGPSGSLQQQYTADTPPPPPPRPAGSRTGQTDGGPPGQQPGVPARPGQPGQGPPQAAGQPPPPPPQQQQQQQQHQQQQ
Ovulgaris_Synshort  (396) MR----------------------------------PPGQQPGVPARPGQPGQGPPQAAGQPPPPPPQQQQQQQQHQQQQ
  Ovulgaris_Syn8.2  (401) MSQGPSGSLQQQYTADTPPPPPPRPAGSRTGQTDGGPPGQQPGVPARPGQPGQGPPQAAGQPPPPPPQQQQQQQQHQQQQ

                          481                                           526
 Obimaculoides_Syn  (476) QSQQQHPHQQQQSGNQAHPPAPGGQGQDEDTMQNLRKTFAGIFGDM
 Ovulgaris_Synlong  (476) -SQQQHPHQQQQSGNQAHPPAPGGQGQDEDTMQNLRKTFAGIFGDM
Ovulgaris_Synshort  (442) -SQQQHPHQQQQSGNQAHPPAPGGQGQDEDTMQNLRKTFAGIFGDM
  Ovulgaris_Syn8.2  (481) -SQQQHPHQQQQSGNQAHPPAPGGQGQDEDTMQNLRKTFAGIFGDM
```

(**h**) Chromatogram of DNA sequences from two clones amplified by ovary and brain using primer 218-219 and sequenced from both ends showing a stop translation codon upstream isoleucine (highlighted in yellow).

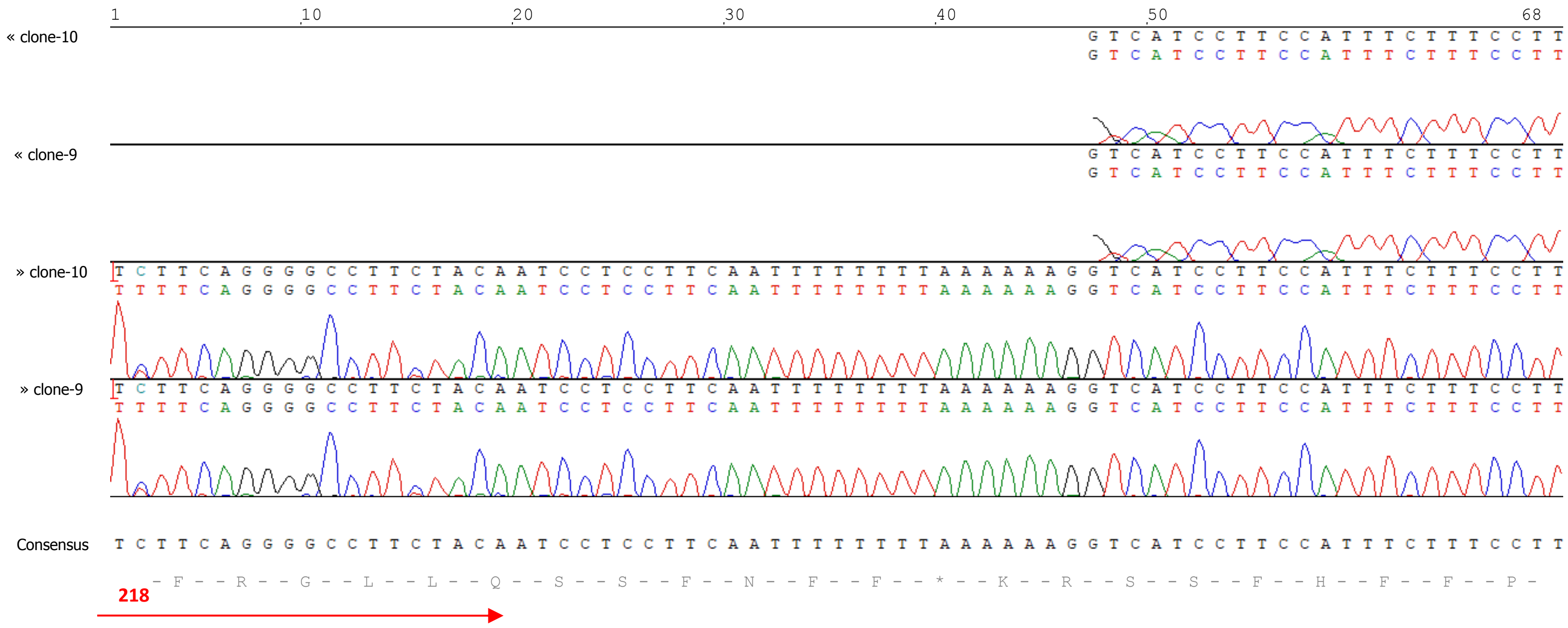

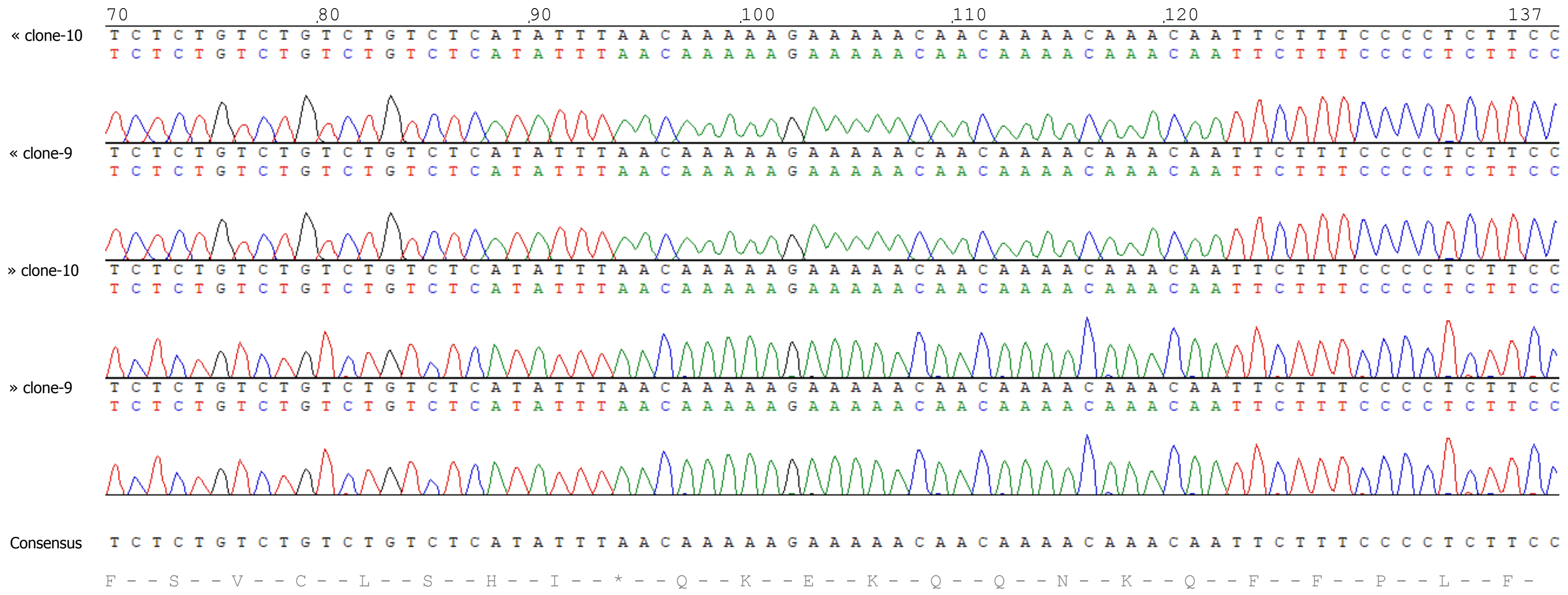
70 80 90 100 110 120 137
« clone-10
« clone-9
» clone-10
» clone-9
T C T C T G T C T G T C T G T C T C A T A T T T A A C A A A A A G A A A A A C A A C A A A A C A A A C A A T T C T T T C C C C T C T T C C
Consensus
T C T C T G T C T G T C T G T C T C A T A T T T A A C A A A A A G A A A A A C A A C A A A A C A A A C A A T T C T T T C C C C T C T T C C
F - - S - - V - - C - - L - - S - - H - - I - - * - - Q - - K - - E - - K - - Q - - Q - - N - - K - - Q - - F - - F - - P - - L - - F -

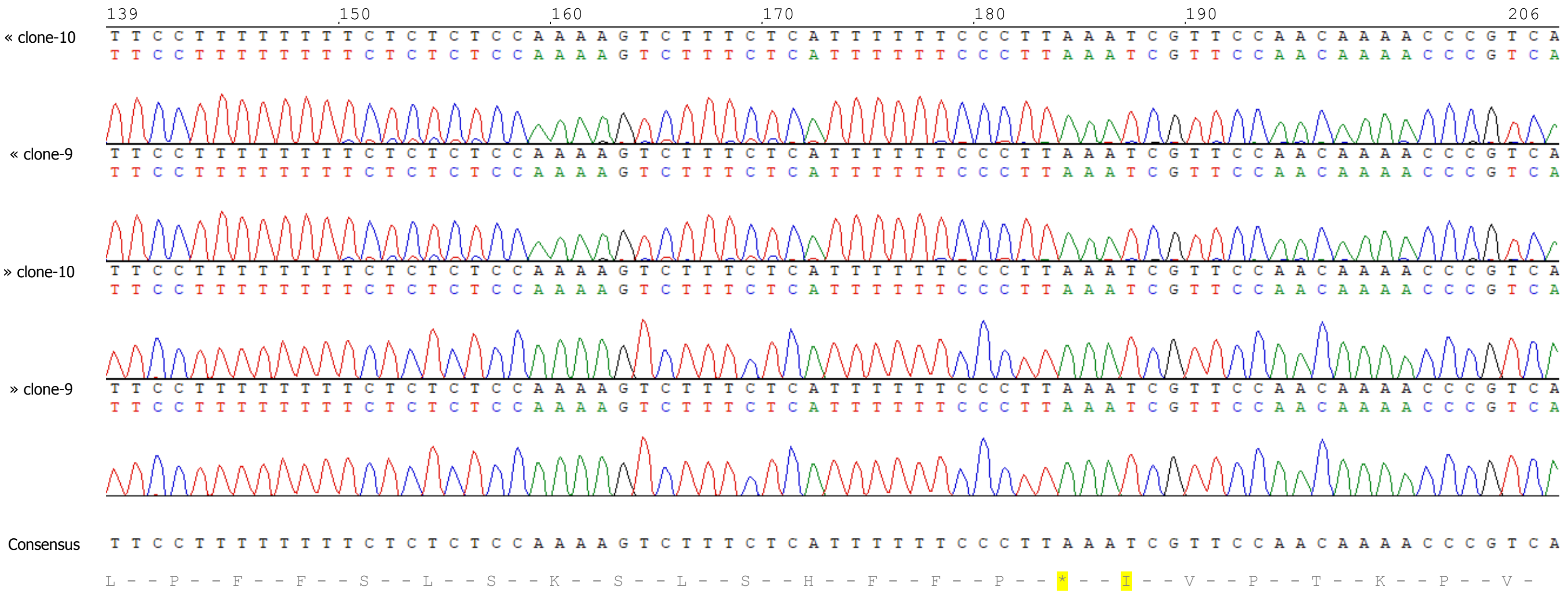
139 150 160 170 180 190 206
« clone-10
« clone-9
» clone-10
» clone-9
T T C C T T T T T T T T C T C T C T C C A A A A G T C T T T C T C A T T T T T T C C C T T A A A T C G T T C C A A C A A A A C C C G T C A
Consensus
T T C C T T T T T T T T C T C T C T C C A A A A G T C T T T C T C A T T T T T T C C C T T A A A T C G T T C C A A C A A A A C C C G T C A
L - - P - - F - - F - - S - - L - - S - - K - - S - - L - - S - - H - - F - - F - - P - - * - - I - - V - - P - - T - - K - - P - - V -

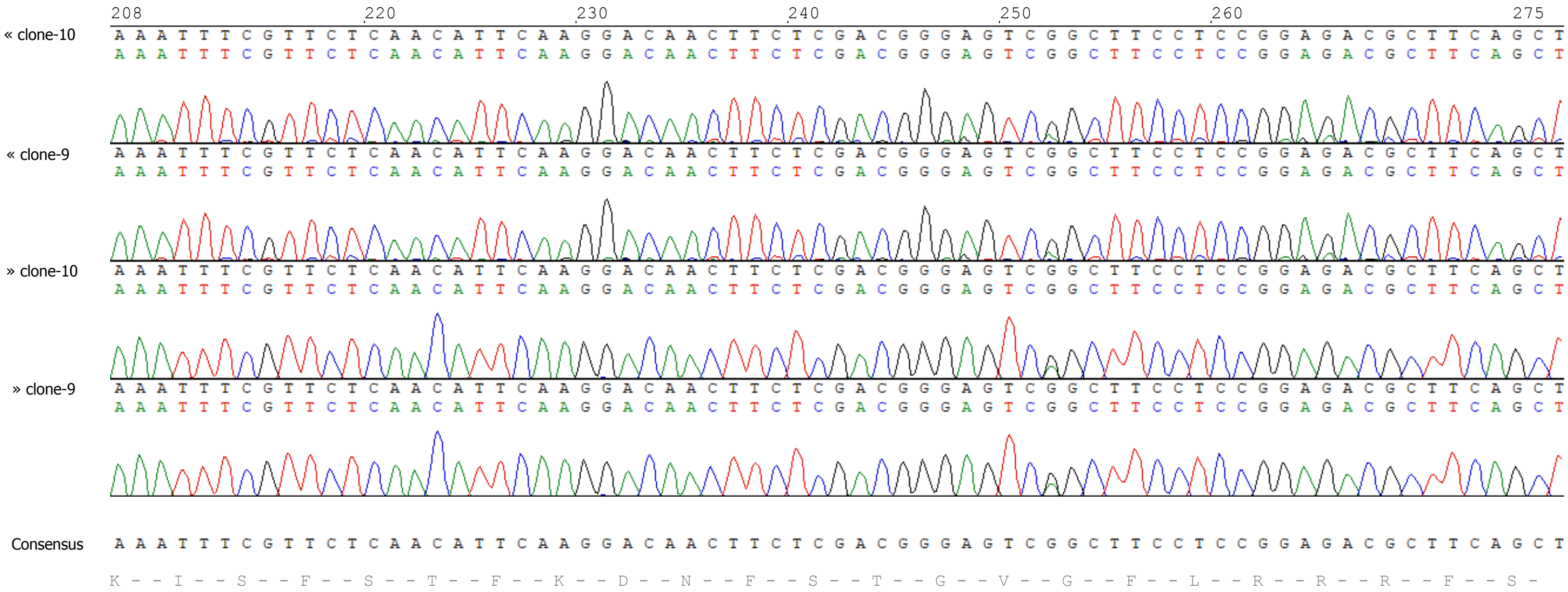
208
220
230
240
250
260
275
« clone-10
« clone-9
» clone-10
» clone-9
Consensus
A A A T T T C G T T C T C A A C A T T C A A G G A C A A C T T C T C G A C G G G A G T C G G C T T C C T C C G G A G A C G C T T C A G C T
K - - I - - S - - F - - S - - T - - F - - K - - D - - N - - F - - S - - T - - G - - V - - G - - F - - L - - R - - R - - R - - F - - S -

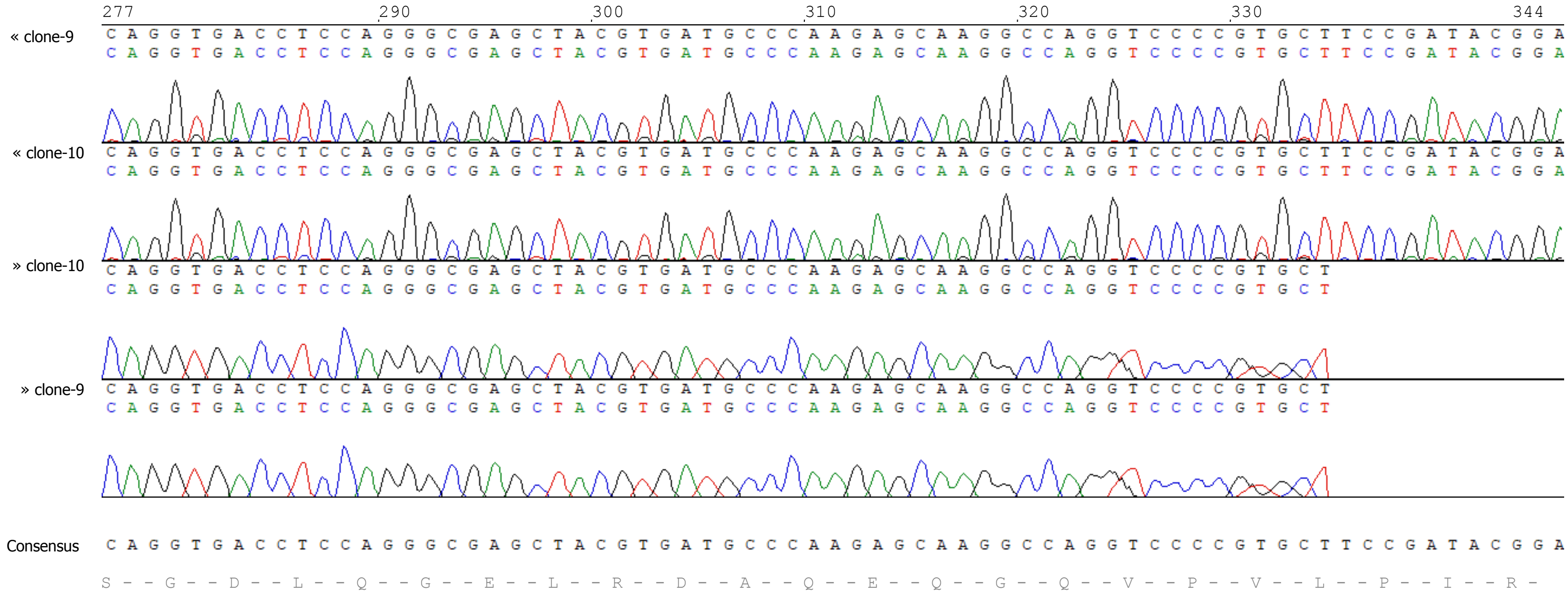
277
290
300
310
320
330
344
« clone-9
« clone-10
» clone-10
» clone-9
Consensus
C A G G T G A C C T C C A G G G C G A G C T A C G T G A T G C C C A A G A G C A A G G C C A G G T C C C C G T G C T T C C G A T A C G G A
S - - G - - D - - L - - Q - - G - - E - - L - - R - - D - - A - - Q - - E - - Q - - G - - Q - - V - - P - - V - - L - - P - - I - - R -

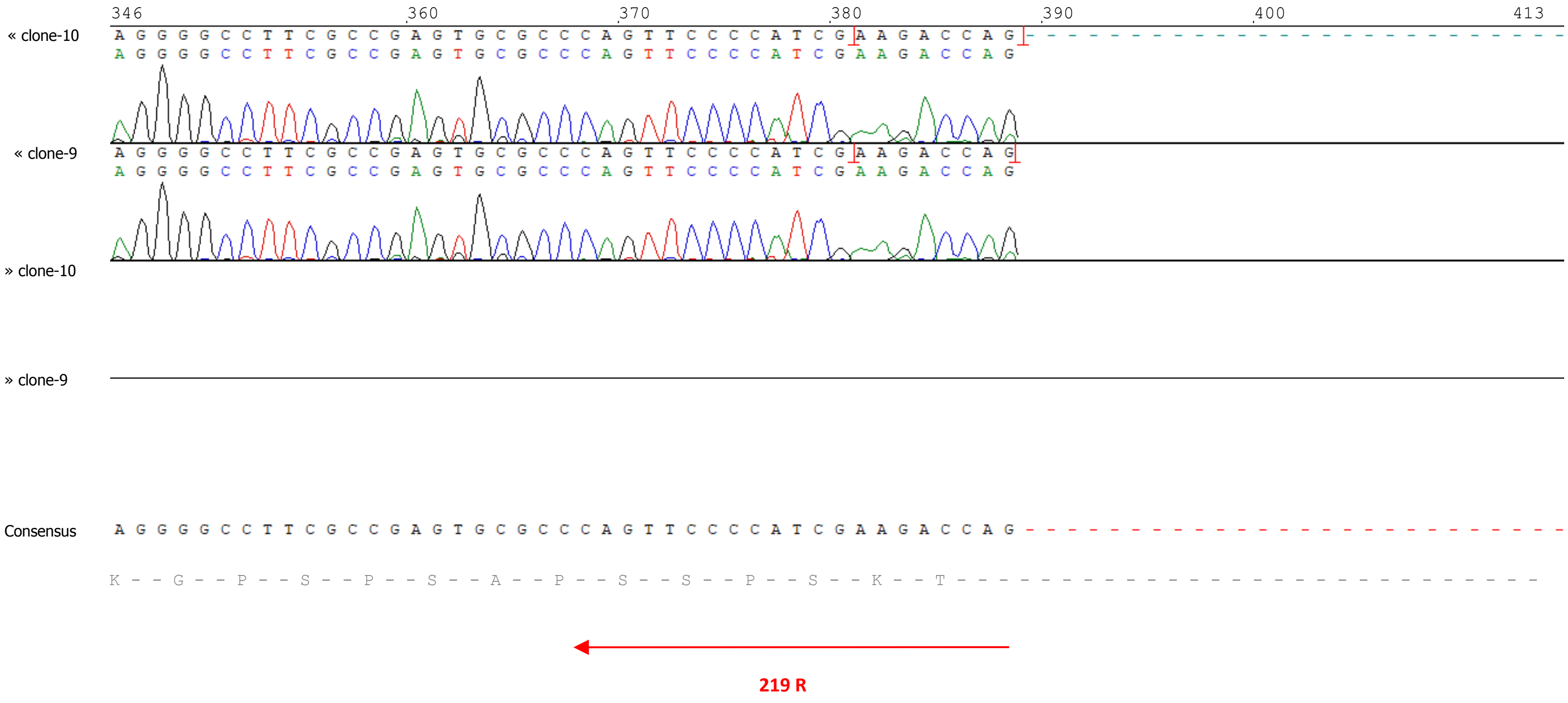
346 360 370 380 390 400 413
« clone-10
A G G G G C C T T C G C C G A G T G C G C C C A G T T C C C C A T C G A A G A C C A G
« clone-9
A G G G G C C T T C G C C G A G T G C G C C C A G T T C C C C A T C G A A G A C C A G
» clone-10
» clone-9
Consensus
A G G G G C C T T C G C C G A G T G C G C C C A G T T C C C C A T C G A A G A C C A G
K - - G - - P - - S - - P - - S - - A - - P - - S - - S - - P - - S - - K - - T
219 R

(**i**) Selected chromatogram files with DNA sequences of the short isoform from clones amplified by PCR with 192-199 primers from ovary, testis and brain are reported. In yellow is shown the region where the long isoform has 102 bp more.

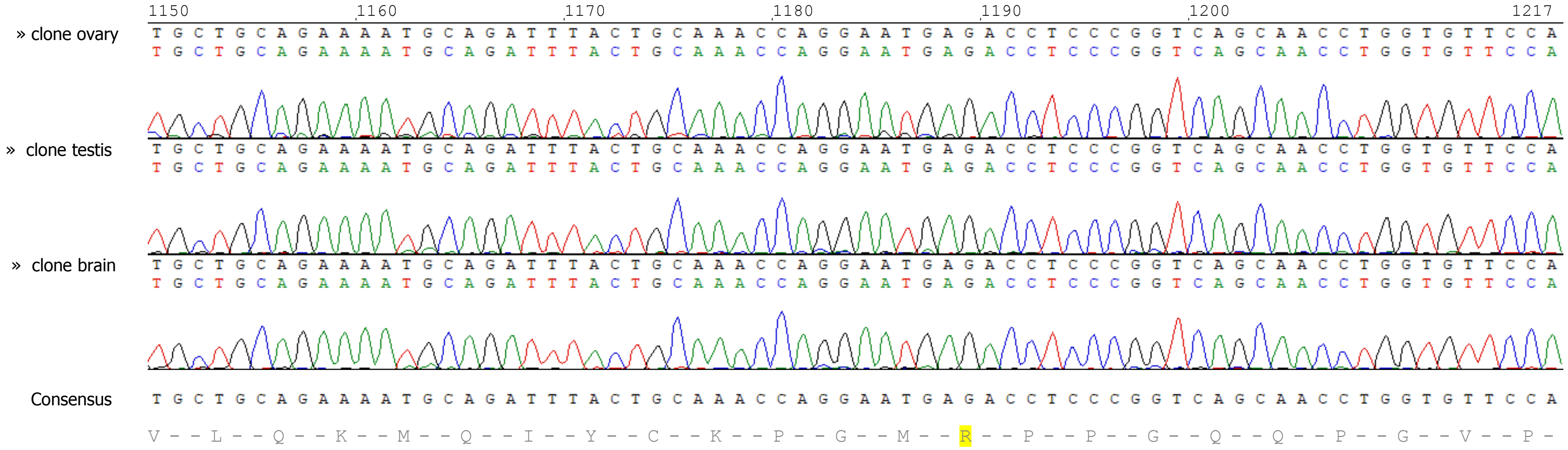

(**j**) Selected chromatogram files with DNA sequences of the long isoform from clones amplified by PCR with 192-199 primers from ovary, testis and brain showing the region where the long isoform has 102 bp more than the short isoform are reported. In yellow and blue are labelled the regions in common and not between the short and long isoforms, respectively.

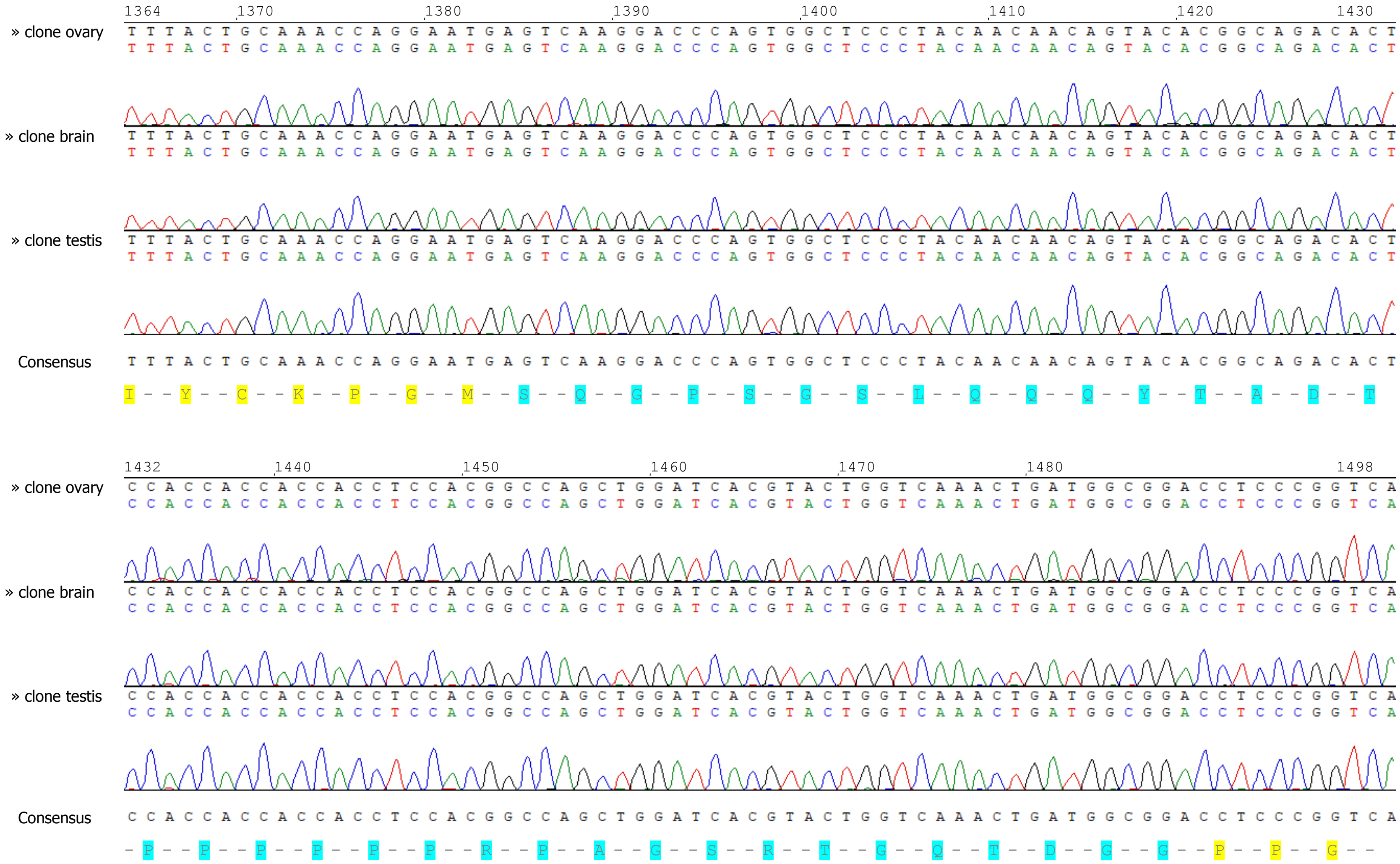

(**k**) A chromatogram file with DNA sequence of clones 17, 20, 15 and 8 amplified by PCR with 192-193 primers. Clone 15 amplified from ovary contains the Syn8.2 isoform showing a stretch of 15 extra nucleotides encoding for 5 amino acids (typical of Syn8.2 isoform and located at the central part of domain C, highlighted in yellow).

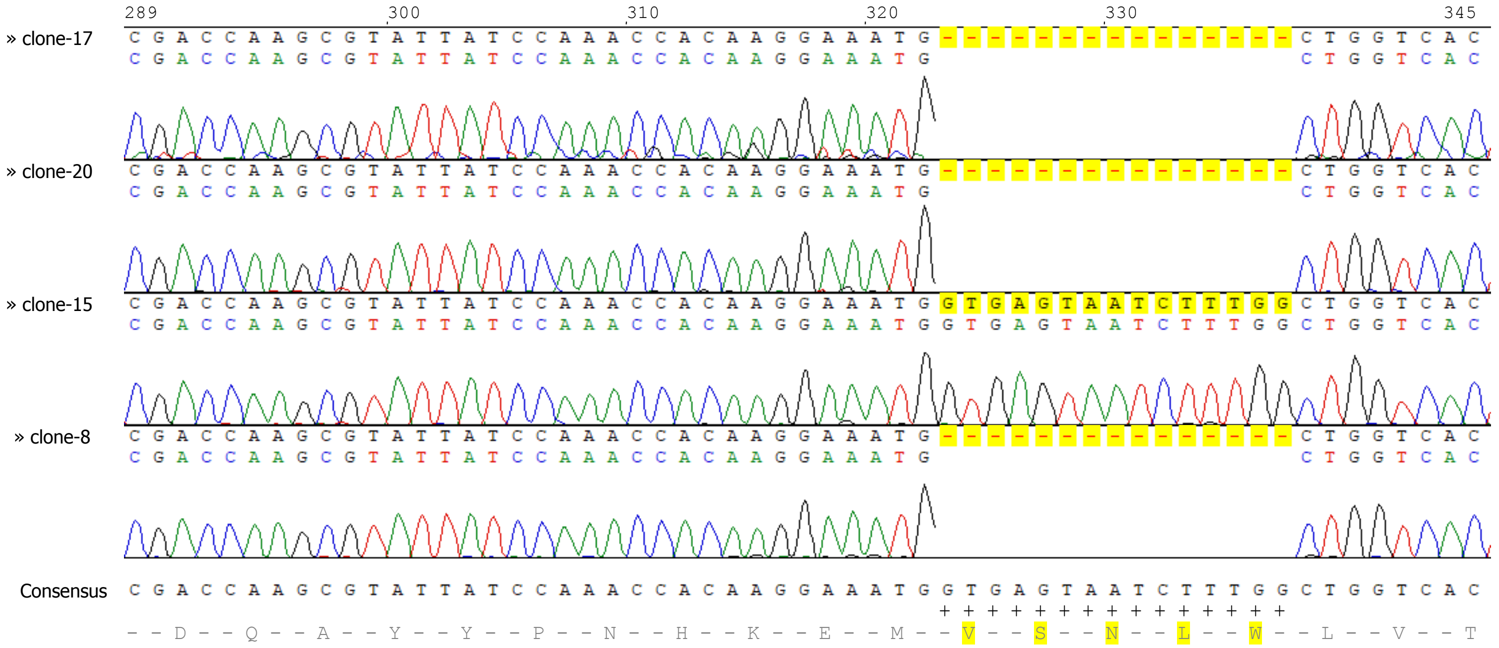

(**I**) Full Contig Assembly of clones 17, 20, 15 and 8 amplified by PCR with 192-193 primers showing the Syn8.2 isoform from clone 15 with the stretch of 15 extra nucleotides encoding for 5 amino acids.

```
          192
          61        70        80        90        100       110       120       130       140       150       160           174
» clone-17  GAATGGCACCAGAGTTGTCAGGTCTTTCAAGCCAGACTTTGTGCTCGTCAGACAACATGTGCGTGACGCTTGTGAGGACTGGCGTAACTTGATCATGGGGTTCCATTATGGCGG
» clone-20  GAATGGCACCAGAGTTGTCAGGTCTTTCAAGCCAGACTTTGTGCTCGTCAGACAACATGTGCGTGACGCTTGTGAGGACTGGCGTAACTTGATCATGGGGTTCCATTATGGCGG
»clone-15   GAATGGCACCAGAGTTGTCAGGTCTTTCAAGCCAGACTTTGTGCTCGTCAGACAACATGTGCGTGACGCTTGTGAGGACTGGCGTAACTTGATCCTGGGGTTCCATTATGGCGG
» clone-8   GAATGGCACCAGAGTTGTCAGGTCTTTCAAGCCAGACTTTGTGCTCGTCAGACAACATGTGCGTGACGCTTGTGAGGACTGGCGTAACTTGATCATGGGGTTCCATTATGGCGG
Consensus   GAATGGCACCAGAGTTGTCAGGTCTTTCAAGCCAGACTTTGTGCTCGTCAGACAACATGTGCGTGACGCTTGTGAGGACTGGCGTAACTTGATCATGGGGTTCCATTATGGCGG
                                                                                                 +
           --N--G--T--R--V--V--R--S--F--K--P--D--F--V--L--V--R--Q--H--V--R--D--A--C--E--D--W--R--N--L--I--M--G--F--H--Y--G--G

          175 180       190       200       210       220       230       240       250       260       270             288
» clone-17  TGTTCCTAGTATTAATTCTATGGATTCCATTTACAACTTTCAAGATAAACCTTGGGTGATGGCACACTTAATCCAGATCCAAAAGAAACTTGGACCGGACAAGTTCCCATTGAT
» clone-20  TGTTCCTAGTATTAATTCTATGGATTCCATTTACAACTTTCAAGATAAACCTTGGGTGATGGCACACTTAATCCAGATCCAAAAGAAACTTGGACCGGACAAGTTCCCATTGAT
» clone-15  TGTTCCTAGTATTAATTCTATGGATTCCATTTACAACTTTCAAGATAAACCTTGGGTGATGGCACACTTAATCCAGATCCAAAAGAAACTTGGACCGGACAAGTTCCCATTGAT
» clone-8   TGTTCCTAGTATTAATTCTATGGATTCCATTTACAACTTTCAAGATAAACCTTGGGTGATGGCACACTTAATCCAGATCCAAAAGAAACTTGGACCGGACAAGTTCCCATTGAT
Consensus   TGTTCCTAGTATTAATTCTATGGATTCCATTTACAACTTTCAAGATAAACCTTGGGTGATGGCACACTTAATCCAGATCCAAAAGAAACTTGGACCGGACAAGTTCCCATTGAT

           --V--P--S--I--N--S--M--D--S--I--Y--N--F--Q--D--K--P--W--V--M--A--H--L--I--Q--I--Q--K--K--L--G--P--D--K--F--P--L--I

          289        300       310       320       330       340       350       360       370       380       390          402
» clone-17  CGACCAAGCGTATTATCCAAACCACAAGGAAATG---------------CTGGTCACTCCAAAATTTCCAGTTGTGGTGAAGATTGGACATGCTCATTCTGGTATGGGAAAGGT
» clone-20  CGACCAAGCGTATTATCCAAACCACAAGGAAATG---------------CTGGTCACTCCAAAATTTCCAGTTGTGGTGAAGATTGGACATGCTCATTCTGGTATGGGAAAGGT
» clone-15  CGACCAAGCGTATTATCCAAACCACAAGGAAATGGTGAGTAATCTTTGGCTGGTCACTCCAAAATTTCCAGTTGTGGTGAAGATTGGACATGCTCATTCTGGTATGGGAAAGGT
» clone-8   CGACCAAGCGTATTATCCAAACCACAAGGAAATG---------------CTGGTCACTCCAAAATTTCCAGTTGTGGTGAAGATTGGACATGCTCATTCTGGTATGGGAAAGGT
 Consensus  CGACCAAGCGTATTATCCAAACCACAAGGAAATG---------------CTGGTCACTCCAAAATTTCCAGTTGTGGTGAAGATTGGACATGCTCATTCTGGTATGGGAAAGGT

                                              ++++++++++++++++
           --D--Q--A--Y--Y--P--N--H--K--E--M--V--S--N--L--W--L--V--T--P--K--F--P--V--V--V--K--I--G--H--A--H--S--G--M--G--K--V
```

```
           403    410          420          430          440          450          460          470          480          490          500              516
» clone-17  GAAGATCGACCACCATCATGCCTTCCAAGACATCTCCAGTGTCGTGGCTGTGACCAAGACGTATGCCACCACAGAGCCACTCATTGACTGCAAGTACGACATCCGTGTTGCAGAA
» clone-20  GAAGATCGACCACCATCATGCCTTCCAAGACATCTCCAGTGTCGTGGCTGTGACCAAGACGTATGCCACCACAGAGCCATTCATTGACTGCAAGTACGACATCCGTGTTGCAGAA
» clone-15  GAAGATCGACCACCATCATGCCTTCCAAGACATCTCCAGTGTCGTGGCTGTGACCAAGACGTATGCCACCACAGAGCCATTCATTGACTGCAAGTACGACATCCGTGTTGCAGAA
»clone-8    GAAGATCGACCACCATCATGCCTTCCAAGACATCTCCAGTGTCGTGGCTGTGACCAAGACGTATGCCACCACAGAGCCATTCATTGACTGCAAGTACGACATCCGTGGGCAGAA
Consensus   GAAGATCGACCACCATCATGCCTTCCAAGACATCTCCAGTGTCGTGGCTGTGACCAAGACGTATGCCACCACAGAGCCATTCATTGACTGCAAGTACGACATCCGTGTTGCAGAA

                                                                                     +                                +
           --K--I--D--H--H--H--A--F--Q--D--I--S--S--V--V--A--V--T--K--T--Y--A--T--T--E--P--F--I--D--C--K--Y--D--I--R--V--Q--K

           517          530          540          550          560          570          580          590          600          610          620      630
» clone-17  GATAGGCAACAATTACAAGGCATTCATTCGCAAATCCATATCAGGAAACTGGAAAGCCAACACCGGGTCAGCAATGCTGGAACAGATAGCTATGAATGACAGATATAAGCTGTG
» clone-20  GATAGGCAACAATTACAAGGCATTCATTCGCAAATCCATATCAGGAAACTGGAAAGCCAACACCGGGTCAGCAATGCTGGAACAGATAGCTATGAATGACAGATATAAGCTGTG
» clone-15  GATAGGCAACAATTACAAGGCATTCATTCGCAAATCCATATCAGGAAACTGGAAAGCCAACACCGGGTCAGCAATGCTGGAACAGATAGCTATGAATGACAGATATAAGCTGTG
» clone-8   GATAGGCAACAATTACAAGGCATTCATTCGCAAATCCATATCAGGAAACTGGAAAGCCAACACCGGGTCAGCAATGCTGGAACAGATAGCTATGAATGACAGATATAAGCTGTG
Consensus   GATAGGCAACAATTACAAGGCATTCATTCGCAAATCCATATCAGGAAACTGGAAAGCCAACACCGGGTCAGCAATGCTGGAACAGATAGCTATGAATGACAGATATAAGCTGTG

           --I--G--N--N--Y--K--A--F--I--R--K--S--I--S--G--N--W--K--A--N--T--G--S--A--M--L--E--Q--I--A--M--N--D--R--Y--K--L--W

           631      640          650          660          670          680          690          700          710          720          730          744
» clone-17  GGTTGATGAGTGCAGCCAAATGTTTGGAGGTCTGGATATTGTAGCTGTGGAAGCCCTGCAGGGTAAAGATGGCCGTGAATATGTCATTGAGGTGAACGACTCATCAATGGTTCT
» clone-20  GGTTGATGAGTGCAGCCAAATGTTTGGAGGTCTGGATATTGTAGCTGTGGAAGCCCTGCAGGGTAAAGATGGCCGTGAATATGTCATTGAGGTGAACGACTCATCAATGGTTCT
» clone-15  GGTTGATGAGTGCAGCCAAATGTTTGGAGGTCTGGATATTGTAGCTGTGGAAGCCCTGCAGGGTAAAGATGGCCGTGAATATGTCATTGAGGTGAACGACTCATCAATGGTTCT
» clone-8   GGTTGATGAGTGCAGCCAAATGTTTGGAGGTCTGGATATTGTAGCTGTGGAAGCCCTGCAGGGTAAAGATGGCCGTGAATATGTCATTGAGGTGAACGACTCATCAATGGTTCT
Consensus   GGTTGATGAGTGCAGCCAAATGTTTGGAGGTCTGGATATTGTAGCTGTGGAAGCCCTGCAGGGTAAAGATGGCCGTGAATATGTCATTGAGGTGAACGACTCATCAATGGTTCT

           --V--D--E--C--S--Q--M--F--G--G--L--D--I--V--A--V--E--A--L--Q--G--K--D--G--R--E--Y--V--I--E--V--N--D--S--S--M--V--L

           745  750          760          770          780          790          800          810          820          830          840              858
» clone-17  GTTAGGGGAGACTCAAGAGGAGGACCGACGCCTCATCTCTGAGATGGTGCTGCAGAAAATGCAGATTTACTGCAAACCAGGAATGAGTCAAGGACCCAGTGGCTCCCTACAACA
» clone-20  GTTAGGGGAGACTCAAGAGGAGGACCGACGCCCCATCTCTGAGATGGTGCTGCAGAAAATGCAGATCTACTGCAAACCAGGAATGAGTCAAGGACCCAGTGGCTCCCTACAACA
» clone-15  GTTAGGGGAGACTCAAGAGGAGGACCGACGCCTCATCTCTGAGATGGTGCTGCAGAAAATGCAGATTTACTGCAAACCAGGAATGAGTCAAGGACCCAGTGGCTCCCTACAACA
» clone-8   GTTAGGGGAGACTCAAGAGGAGGACCGACGCCTCATCTCTGAGATGGTGCTGCAGAAAATGCAGATTTACTGCAAACCAGGAATGAGTCAAGGACCCAGTGGCTCCCTACAACA
Consensus   GTTAGGGGAGACTCAAGAGGAGGACCGACGCCTCATCTCTGAGATGGTGCTGCAGAAAATGCAGATTTACTGCAAACCAGGAATGAGTCAAGGACCCAGTGGCTCCCTACAACA

                                            +                                +        +
           --L--G--E--T--Q--E--E--D--R--R--L--I--S--E--M--V--L--Q--K--M--Q--I--Y--C--K--P--G--M--S--Q--G--P--S--G--S--L--Q--Q
```

```
           799        810       820       830       840       850       860       870       880
» clone-17 ACAGTACACGGCAGACACTCCACCACCACCACCTCCACGGCCAGCTGGATCACGTACTGGTCAA-CTGATGGCGGACCTCC-
»clone-20  ACAGTACACGGCAGACACTCCACCACCGCCACCTCCACGGCCAGCTGGATCACGTACTGGTCAAACTGATGGCGGACCTCC
»clone-15  ACAGTACACGGCAGACACTCCACCACCACCACCTCCACGGCCAGCTGGATCACGCACTGGTCAAACTGATGGCGGACCTCC
»clone-8   ACAGTACACGGCAGACACTCCACCACCACCACCTCCACGGCCAGCTGGATCACGTACTGGTCAAACTGATGGCGGACCTCCC
Consensus  ACAGTACACGGCAGACACTCCACCACCACCACCTCCACGGCCAGCTGGATCACGTACTGGTCAAACTGATGGCGGACCTCCC

                                     +                                   +            +                        +
         --C--Y--T--A--D--T--P--P--P--P--P--P--R--P--A--G--S--R--T--G--Q--T--D--G--G--P--P--------------------------------

                                                                                        193
```
