# Supplementary material for: Synapsins are expressed at neuronal and non-neuronal locations in Octopus vulgaris
Source: Sci Rep. 2019 Oct 28;9:15430. doi: 10.1038/s41598-019-51899-y (PMC6817820; doi:10.1038/s41598-019-51899-y)
Supplement: Supplementary file 2 — Additional file 2 [file 41598_2019_51899_MOESM2_ESM.pdf]

Synapsins are expressed at neuronal and non-neuronal locations in Octopus vulgaris

Federica Maiole[1,2+], Giulia Tedeschi[2,3+], Simona Candiani[4*], Luca Maragliano[1,5], Fabio Benfenati[1,5], Letizia Zullo[1,5*]

Supplementary Info:
Additional File 2: List of sequences with the corresponding accession numbers used in this work.

Sequences used for sequence and phylogenetic analysis		
**Name of sequence**	**Species**	**GenBank accession number**
SynIa	*Homo sapiens*	NP_008881
SynIIa	*Homo sapiens*	NP_598328
SynIIb	*Homo sapiens*	NP_003169
SynIIIa	*Homo sapiens*	NP_003481
SynIIa	*Rattus norvegicus*	NP_001029192
Syn	*Branchiostoma floridae*	ACT32024
Syn2.1	*Aplysia californica*	AAK83048
Syn7.1	*Aplysia californica*	AAK83049
Syn8.2	*Aplysia californica*	AAK83050
Syn11.1	*Aplysia californica*	AAK83047
Syn	*Helix pomatia*	AAS45543
Synlong	*Loligo pealei*	AAC24823
Synshort	*Loligo pealei*	AAC24822
Syn	*Octopus bimaculoides*	XP_014784838
Synlong	*Octopus vulgaris*	KY768851
Synshort	*Octopus vulgaris*	KY768852
Syn8.2	*Octopus vulgaris*	KY768853
Syn	Drosophila melanogaster	NP_731457.2
