# Supplementary material for: Synapsins are expressed at neuronal and non-neuronal locations in Octopus vulgaris
Source: Sci Rep. 2019 Oct 28;9:15430. doi: 10.1038/s41598-019-51899-y (PMC6817820; doi:10.1038/s41598-019-51899-y)
Supplement: Supplementary file 3 — Additional file 3 [file 41598_2019_51899_MOESM3_ESM.pdf]

Synapsins are expressed at neuronal and non-neuronal locations in Octopus vulgaris

Federica Maiole[1,2+], Giulia Tedeschi[2,3+], Simona Candiani[4*], Luca Maragliano[1,5], Fabio Benfenati[1,5], Letizia Zullo[1,5*]

Supplementary Info:
Additional File 3: Sequence identity matrix of synapsin domains of mammals and O. vulgaris synapsins.

Domain A

Percent Identity (upper) / Divergence (lower)

	1	2	3	4	5	6		
1		86.7	86.7	86.7	86.7	20.0	1	HomosapiensSynIa
2	14.7		100.0	90.0	100.0	19.4	2	HomosapiensSynIIa
3	14.7	0.0		90.0	100.0	19.4	3	HomosapiensSynIIb
4	14.7	10.8	10.8		90.0	16.7	4	HomosapiensSynIIIa
5	14.7	0.0	0.0	10.8		19.4	5	RattusnorvegicusSynIIA
6	246.0	254.0	254.0	296.0	254.0		6	OctopusvulgarisSynlong
	1	2	3	4	5	6		

Domain B

Percent Identity (upper) / Divergence (lower)

	1	2	3	4	5	6		
1		40.4	41.3	29.8	37.5	25.0	1	HomosapiensSynIa
2	78.0		99.0	84.6	40.4	24.0	2	HomosapiensSynIIa
3	74.4	1.2		83.7	40.4	25.0	3	HomosapiensSynIIb
4	102.0	12.1	13.5		38.5	22.1	4	RattusnorvegicusSynIIa
5	133.0	86.9	86.9	84.6		42.3	5	HomosapiensSynIIIa
6	233.0	191.9	169.5	185.4	140.9		6	OctopusvulgarisSynlong
	1	2	3	4	5	6		

Domain C

Percent Identity (upper) / Divergence (lower)

	1	2	3	4	5	6	7		
1		77.2	77.2	68.8	77.8	61.9	59.7	1	HomosapiensSynIa
2	26.4		100.0	72.5	97.2	66.9	64.7	2	HomosapiensSynIIa
3	26.4	0.0		72.5	97.2	66.9	64.7	3	HomosapiensSynIIb
4	39.8	35.2	35.2		72.5	63.4	61.6	4	HomosapiensSynIIIa
5	25.5	3.0	3.0	35.2		65.9	64.1	5	RattusnorvegicusSynIIa
6	48.5	41.1	41.1	47.3	42.8		96.9	6	OctopusvulgarisSynlong
7	50.8	43.2	43.2	48.9	44.3	1.7		7	OctopusvulgarisSyn8.2
	1	2	3	4	5	6	7		

Domain D

Percent Identity (upper) / Divergence (lower)

	1	2	3	4	5	6	7		
1		18.2	11.3	16.9	17.7	21.2	15.6	1	HomosapiensSynIa
2	152.3		69.7	46.3	93.1	51.9	55.4	2	HomosapiensSynIIa
3	143.9	34.4		50.2	68.0	60.2	63.6	3	HomosapiensSynIIb
4	146.8	197.0	199.0		46.3	55.4	52.4	4	HomosapiensSynIIIa
5	161.1	11.7	40.0	193.5		51.9	55.4	5	RattusnorvegicusSynIIa
6	76.7	243.0	246.0	156.9	229.0		84.8	6	OctopusvulgarisSynlong
7	55.7	204.0	163.8	121.4	193.5	2.1		7	OctopusvulgarisSynshort
	1	2	3	4	5	6	7		

Domain E

Percent Identity (upper) / Divergence (lower)

	1	2	3	4	5		
1		76.5	66.7	76.5	31.4	1	HomosapiensSynIa
2	29.0		74.5	100.0	25.5	2	HomosapiensSynIIa
3	42.7	29.7		74.5	27.5	3	HomosapiensSynIIIa
4	29.0	0.0	29.7		25.5	4	RattusnorvegicusSynIIa
5	107.7	140.9	140.9	140.9		5	OctopusvulgarisSynlong
	1	2	3	4	5		
