# Supplementary material for: Synapsins are expressed at neuronal and non-neuronal locations in Octopus vulgaris
Source: Sci Rep. 2019 Oct 28;9:15430. doi: 10.1038/s41598-019-51899-y (PMC6817820; doi:10.1038/s41598-019-51899-y)
Supplement: Supplementary file 4 — Additional file 4 [file 41598_2019_51899_MOESM4_ESM.pdf]

Synapsins are expressed at neuronal and non-neuronal locations in Octopus vulgaris

Federica Maiole[1,2+], Giulia Tedeschi[2,3+], Simona Candiani[4*], Luca Maragliano[1,5], Fabio Benfenati[1,5], Letizia Zullo[1,5*]

Supplementary Info:
Additional File 4: A multi-alignment in Fasta format used to reconstruct the phylogenetic tree.

>HomosapiensSynIa_NP_008881
------------------------MNYLRRRLS--DSNFMANLPNGYMTDLQRPQP----
PPPPPGAHSPGATPGPGTATAERSSGVAP--AASPAA------PSP--GSSGGGGFFSSL
SNAVKQT-TAAAAATFSEQVGGGSGGAGRGGAASR----VLLVIDEPHTDWAKYFKGKKI
HGGIDIKVEQAEFSDLNLVAHANGGFSVDMEVLRNGVKVV-RSLKPDFVLIRQHAFSMAR
NGDYRSLVIGLQYAGIPSVNSLHSVYNFCDKPWVFAQMVRLHKKLGTEEFPLIDQTFYPN
HKEMLS-----STTYPVVVKMGHAHSGMGKVKVDNQHDFQDIASVVALTKT----YATAE
PFIDAKYDVRVQKIGQNYKAYMRTSVSGNWKTNTGSAMLEQIAMSDRYKLWVDTCSEIFG
GLDICAVEALHGKDGRDHIIEVVGSSMPLIGDHQDEDKQLIVELVVNKMAQALPRQRQRD
ASPGRGSHGQTPSPGALPLGRQTSQQPAGPPAQQRPPPQGGPPQPGPGPQRQGPPLQQRP
PPQG-QQHLSGLGPPAGSPLPQRLPSPTSAPQQPASQAAPPTQGQGRQSRPVAGGPGAPP
AARPPASPSPQRQAGPPQATRQTSVSGPAPPKASGAPPGGQQRQGPPQKPPGPAGPTRQA
SQAGPVPRTGPPTTQQPRPSGPGPAGAPKPQLAQKPSQDVPPPATAAAGGPPHPQLNKSQ
SLTNAFNLPEPAPPRPSLS---QDEVKAETIRSLRKSFASLFSD-

>HomosapiensSynIIa_NP_598328
-----------------------MMNFLRRRLS--DSSFIANLPNGYMTDLQRPEPQQPP
PPPPPGPGAASASAAPPTASPGPERRPPP---ASAPAPQPAPTPS------VGSSFFSSL
SQAVKQT-AASAGLVDAPAP---APAAAR---KAK----VLLVVDEPHADWAKCFRGKKV
LGDYDIKVEQAEFSELNLVAHADGTYAVDMQVLRNGTKVV-RSFRPDFVLIRQHAFGMAE
NEDFRHLIIGMQYAGLPSINSLESIYNFCDKPWVFAQLVAIYKTLGGEKFPLIEQTYYPN
HKEMLT-----LPTFPVVVKIGHAHSGMGKVKVENHYDFQDIASVVALTQT----YATAE
PFIDSKYDIRVQKIGNNYKAYMRTSISGNWKTNTGSAMLEQIAMSDRYKLWVDTCSEMFG
GLDICAVKAVHGKDGKDYIFEVMDCSMPLIGEHQVEDRQLITELVISKMNQLLSR-----
------------TPALSPQRPLTTQQ-----------PQSGTLK-DPDSSKTPP---QRP
PPQGGPGQPQGMQPPG-----KVLPP-----------------------RRLPPGPSLPP
SSSSSSSSS----SSAPQRPGGPTAHGDAPSSSSSLAEAQPPLAAPPQKPQ---------
---PHPQLNKSQ
SLTNAFSFSESSFFRSSAN---EDEAKAETIRSLRKSFASLFSD-

>HomosapiensSynIIb_NP_003169
-----------------------MMNFLRRRLS--DSSFIANLPNGYMTDLQRPEPQQPP
PPPPPGPGAASASAAPPTASPGPERRPPP---ASAPAAQPAPTPS------VGSSFFSSL
SQAVKQT-AASAGLVDAPAP---APAAAR---KAK----VLLVVDEPHADWAKCFRGKKV
LGDYDIKVEQAEFSELNLVAHADGTYAVDMQVLRNGTKVV-RSFRPDFVLIRQHAFGMAE
NEDFRHLIIGMQYAGLPSINSLESIYNFCDKPWVFAQLVAIYKTLGGEKFPLIEQTYYPN
HKEMLT-----LPTFPVVVKIGHAHSGMGKVKVENHYDFQDIASVVALTQT----YATAE
PFIDSKYDIRVQKIGNNYKAYMRTSISGNWKTNTGSAMLEQIAMSDRYKLWVDTCSEMFG
GLDICAVKAVHGKDGKDYIFEVMDCSMPLIGEHQVEDRQLITELVISKMNQLLSR-----
------------TPALSPQRPLTTQQ-----------PQSGTLK-DPDSSKTPP---QRP
PPQG------CLQ--------YILDC-----------------------NGIAVGPKQVQ
AS--

>HomosapiensSynIIIa_NP_003481
------------------------MNFLRRRLS--DSSFMANLPNGYMTDLQRPDS----
----------STSS--PASPAMERRHPQP-LAASFS--------SP------GSSLFSSL
SSAMKQAPQATSGLMEPPGPS--TPIVQR----PR----ILLVIDDAHTDWSKYFHGKKV
NGEIEIRVEQAEFSELNLAAYVTGGCMVDMQVVRNGTKVVSRSFKPDFILVRQHAYSMAL
GEDYRSLVIGLQYGGLPAVNSLYSVYNFCSKPWVFSQLIKIFHSLGPEKFPLVEQTFFPN
HKPMVT-----APHFPVVVKLGHAHAGMGKIKVENQLDFQDITSVVAMAKT----YATTE
AFIDSKYDIRIQKIGSNYKAYMRTSISGNWKANTGSAMLEQVAMTERYRLWVDSCSEMFG
GLDICAVKAVHSKDGRDYIIEVMDSSMPLIGEHVEEDRQLMADLVVSKMSQLPMPG----
------------GTAPSPLRPWAPQIKS--------AKSPGQAQLGPQLGQPQP----RP
PPQGGPRQAQSPQPQR-----SGSPS-----------------------QQRLSPQGQQP
LSPQSGSPQQQRSPGSPQLSRASSGSSPNQASKPGATLASQPRPPVQGRSTSQQGEESKK
--PAPPHPHLNKSQ
SLTNSLSTSDTSQ-RGTPS---EDEAKAETIRNLRKSFASLFSD-

>BranchiostomafloridaeSyn_ACT32024
------------------------MNYLRRRFS--DTNIAANLPNGYLSGL-GGEDD---
----------KNQQ-QPAA-------APP-RGTS--A-----PSSP--------------
ARSMPPG--GMAGGRDGERN------TTK----------TLLVIDDQHTDWGKYFRGKRI
NNEYEIRVEQADFSEINLAAYSDTGTMIDMQINRQGTKVV-RSFRPDFVLVRQHCRGLDA
NQDYRSVILGLRWGAIPSVNTLLSIYNFMEKPWVYAHLLQIRKRIGKEKFPLIDRAYYPN
HKEMLI-----TPKFPVVVKIGHAHAGLGKVKVENHHDFQDIASVVAVANT----YATTE
PFIDAKHDIRVQKIGNNYKAYMRTSISGNWKANTGSAMLEQIPMTEKYRVWVDAVSEIFG

```
GLDICAVEAIHGKDGKDYIIEVNDSTMPLLGENQEEDRQLISDVVLQRMTQVCRAG----
------------ASAAQNINPHNAWR-------------RVLVMSGLSTDAEP-----A
EPQT---------------------------------------------------SAKSMTG
FLNLGGSAS-----APPSASSSHSTLPGAQAGAAGPPP---------------------
------------------------------------------------------------
----------------------ADEDQAESIRKLRKAFSGIFGEP

>Loligopealeisynlong_AAC24823
FFFDLFFQTAKISFSGFRDNFSTGVGFLKRRFSSGDLQGE--LRDAQEQGQVPILP----
--------------------------IRK--GPSPSA-----PSSP---------SK-TT
AAGIAKGVFSG-----PKASVNKD----R----CK----TLLVIDDPHTDWSKYFRGKKI
FGDWDLRVEQVEFHEINLAAYTDQGTMVDIQVMRNGTRVV-RSFKPDFVLVRQHVR--DA
SEDWRNLIMGFHYGGIPSLNSFHSIYNFQDKPWTMAHLIQIQKKVGAENFPLIEQAYYPN
HKEML-----VTPKFPVVVKIGHAHSGMGKVKVDHHHAFQDIASVVAVTKS----YATTE
PYIDCKCDIHVQKIGNNYKAFLRKSISGNWKANTGSAMLEQIQMNEKYKLWVDECSQLFG
GLDIVAVEALQGKDGREYIIEVNDSSMALLGETQEEDRRLIAEMVLQKMHMYCKPNT---
------------MSQAMSSGTIQSA----------------------------------A
DSTA-TP--------------------------------------------PPPPPRPA
SSRPPPPPE---SGGPPSQPPPRAPGRQMPPGQG-PP----------------------
--------------------------------------------------PGHPSQVPP-
--------GQGPPQPMTSMGQSQDED---TMQNLRKTFAGIFGDV

>LoligopealeiSynshort_AAC24822
FFFDLFFQTAKISFSGFRDNFSTGVGFLKRRFSSGDLQGE--LRDAQEQGQVPILP----
--------------------------IRK--GPSPSA-----PSSP---------SK-TT
AAGIAKGVFSG-----PKASVNKD----R----CK----TLLVIDDPHTDWSKYFRGKKI
FGDWDLRVEQVEFHEINLAAYTDQGTMVDIQVMRNGTRVV-RSFKPDFVLVRQHVR--DA
SEDWRNLIMGFHYGGIPSLNSFHSIYNFQDKPWTMAHLIQIQKKVGAENFPLIEQAYYPN
HKEML-----VTPKFPVVVKIGHAHSGMGKVKVDHHHAFQDIASVVAVTKS----YATTE
PYIDCKCDIHVQKIGNNYKAFLRKSISGNWKANTGSAMLEQIQMNEKYKLWVDECSQLFG
GLDIVAVEALQGKDGREYIIEVNDSSMALLGETQEEDRRLIAEMVLQKMHMYCKPNT---
------------MS----------------------------------------------
------------------------------------------------------------
--------------GPPSQPPPRAPGRQMPPGQG-PP----------------------
--------------------------------------------------PGHPSQVPP-
--------GQGPPQPMTSMGQSQDED---TMQNLRKTFAGIFGDV

>OctopusbimaculoidesSyn_XP_014784838
---IVPTKPVKISFSTFKDNFSTGVSFLRRRFSSGDLQGE--LRDAQEQGQVPVLP----
--------------------------IRK--GPSPSA-----PSSP---------SK-TS
AVGIAKGIFSG-----QRSNVNKD----R----CK----TLLVIDDPHTDWSKYFRGKKL
FGDWDIRIEQAEFPEINVAAYTDQGTMVDIQVLRNGTRVV-RSFKPDFVLVRQHVR--DA
CEDWRNLIMGFHYGGVTSINSMDSIYNFQDKPWVMAHLIQIQKKLGPDKFPLIDQAYYPN
HKEML-----VTPKFPVVVKIGHAHSGMGKVKIDHHHAFQDISSVVAVTKT----YATTE
PFIDCKYDIRVQKIGNNYKAFIRKSISGNWKANTGSAMLEQIAMNDRYKLWVDECSQMFG
GLDIVAVEALQGKDGREYIIEVNDSSMVLLGETQEEDRRLISEMVLQKMQIYCKPG----
------------MNQG-PSGSLQPQYTA---------DTPPPPPPPRPAGSRTGQT---DG
GPPG-QQ--------------------------------------------PGVPARPG
QPG-QGPPQA--AGQPPPPPPPQQQQQQQQHQQQQQSQ----------------------
--------------------------------------------------QQHPHQQQQ-
--------SGNQAHPPAPGGQGQDED---TMQNLRKTFAGIFGDM

>OctopusvulgarisSynlong_KY768851
---IVPTKPVKISFSTFKDNFSTGVGFLRRRFSSGDLQGE--LRDAQEQGQVPVLP----
--------------------------IRK--GPSPSA-----PSSP---------SK-TS
AVGIAKGIFSG-----QRSNVNKD----R----CK----TLLVIDDPHTDWSKYFRGKKL
FGDWDIRIEQAEFPEINVAAYTDQGTMVDIQVLRNGTRVV-RSFKPDFVLVRQHVR--DA
CEDWRNLIMGFHYGGVPSINSMDSIYNFQDKPWVMAHLIQIQKKLGPDKFPLIDQAYYPN
HKEML-----VTPKFPVVVKIGHAHSGMGKVKIDHHHAFQDISSVVAVTKT----YATTE
PFIDCKYDIRVQKIGNNYKAFIRKSISGNWKANTGSAMLEQIAMNDRYKLWVDECSQMFG
GLDIVAVEALQGKDGREYVIEVNDSSMVLLGETQEEDRRLISEMVLQKMQIYCKPG----
------------MSQG-PSGSLQQQYTA---------DTPPPPPPPRPAGSRTGQT---DG
GPPG-QQ--------------------------------------------PGVPARPG
QPG-QGPPQA--AGQPPPPPPPQQQQQQQQHQQQQ-SQ----------------------
--------------------------------------------------QQHPHQQQQ-
--------SGNQAHPPAPGGQGQDED---TMQNLRKTFAGIFGDM

>OctopusvulgarisSynshort_KY768852
```

---IVPTKPVKISFSTFKDNFSTGVGFLRRRFSSGDLQGE--LRDAQEQGQVPVLP----
--------------------------IRK--GPSPSA-----PSSP---------SK-TS
AVGIAKGIFSG-----QRSNVNKD----R----CK----TLLVIDDPHTDWSKYFRGKKL
FGDWDIRIEQAEFPEINVAAYTDQGTMVDIQVLRNGTRVV-RSFKPDFVLVRQHVR--DA
CEDWRNLTMGFHYGGVPSINSMDSIYNFQDKPWVMAHLIQIQKKLGPDKFPLIDQAYYPN
HKEML-----VTPKFPVVVKIGHAHSGMGKVKIDHHHAFQDISSVVAVTKT----YATTE
PFIDCKYDIRVQKIGNNYKAFIRKSISGNWKANTGSAMLEQIAMNDRYKLWVDECSQMFG
GLDIVAVEALQGKDGREYVIEVNDSSMVLLGETQEEDRRLISEMVLQKMQIYCKPG----
------------MR--
-PPG-QQ--PGVPARPG
QPG-QGPPQA--AGQPPPPPPQQQQQQQHQQQQ-SQ----------------------
--QQHPHQQQQ-
--------SGNQAHPPAPGGQGQDED---TMQNLRKTFAGIFGDM

>OctopusvulgarisSyn8.2_KY768853
---IVPTKPVKISFSTFKDNFSTGVGFLRRRFSSGDLQGE--LRDAQEQGQVPVLP----
--------------------------IRK--GPSPSA-----PSSP---------SK-TS
AVGIAKGIFSG-----QRSNVNKD----R----CK----TLLVIDDPHTDWSKYFRGKKL
FGDWDIRIEQAEFPEINVAAYTDQGTMVDIQVLRNGTRVV-RSFKPDFVLVRQHVR--DA
CEDWRNLIMGFHYGGVPSINSMDSIYNFQDKPWVMAHLIQIQKKLGPDKFPLIDQAYYPN
HKEMVSNLWLVTPKFPVVVKIGHAHSGMGKVKIDHHHAFQDISSVVAVTKT----YATTE
PFIDCKYDIRVQKIGNNYKAFIRKSISGNWKANTGSAMLEQIAMNDRYKLWVDECSQMFG
GLDIVAVEALQGKDGREYVIEVNDSSMVLLGETQEEDRRLISEMVLQKMQIYCKPG----
------------MSQG-PSGSLQQQYTA---------DTPPPPPPRPAGSRTGQT---DG
GPPG-QQ--PGVPARPG
QPG-QGPPQA--AGQPPPPPPQQQQQQQHQQQQ-SQ----------------------
--QQHPHQQQQ-
--------SGNQAHPPAPGGQGQDED---TMQNLRKTFAGIFGDM

>HelixpomatiaSyn_AAS45543
-----------------------MNFLRRRFSSGDLQGE--ANEKEDPPNVGILN----
--------------------------FKK--GPSPSA-----PNSP---------SKSAS
PATIGQKLFSG---TVGVKPVSKD----R----YK----TLLVIDGQHTDWSKYFKGKKL
FGDWDVKVEQAEFSELNLASNSETGTTVEIQAIRNGNKTT-RSLKPDFLLIRQHVR--DA
KVDWRHLLLGFRYGGVPSINSLTAEFNFLDKPWVFAQLIDIQKRLSKDVFPLIDQTYFSN
HEEML-----NSPKFPLVVKIGHAHRGLGKIKVDNVQTLEDLASVMATMSS----YATTE
PFIDSKYDIHVQKIGTNYKAYLRKSIAGNWKANTGSAMLEQIPMDERFKLWADECSQLFG
GLDVVSVEAIQGKDGRDHIIEVNGSSMALLGEAQEEDRRLISEMVMAKMQMMCKPA----
---------QQPLSKASSSQSITPQANG---------AQKPVL----AASPSRQ------
-AQG--RP---LDTSAQAT
PGQARGPPS---SGGLPGVSNSQTPLSNQPSHLS-NP---------------------
--PPQPFPT---
--------STSGPQGLPRMASKDEED---TMKNLRKTFAGIFGDV

>AplysiacalifornicaSyn8.2_AAK83050
-----------MSFSNFKDSFGSGMNYLRRRFSSGDLQGE--ASDNDDSPNVGGLN----
--------------------------FRK--GPSPSA-----PNSP---------SKSAS
SANLGQRLFSSSSSSSGKPSYNKD----R----CK----TLLVIDDQHTDWSKYFRGKKL
FGDWDVRVEQAEFSELNLAAYSDSGTMVDIQVTRNGTKVV-RSFKPDFVLIRQHVR--DA
HEDWRNLLLGFKYGAIPSVNSLTAEYNFLDKPWVFAQLIEIQKRLGKESFPLIDQAYYPN
HKEMVSNMWLITPKFPVVVKIGHAHSGLGKIKIDTVHGFQDMASIVAVTSS----YATTE
PFVDSKYDIHVQKIGTNYKAYLRKSISGNWKANTGSAMLEQIAMNERFKLWVDECSQLFG
GLDVVAVEAIHGKDGREHIIEVNGSSMTLLGEAQEEDRRLIAELVLAKMQAMCKPV----
---------QTSMSKATSSGAIMHQVNG---------SHSGPQ----AGLRSSH------
-APR--KP---GQGRGHDG
GPPPQGPMR---APGMPGGPPAPVPR---PRHMN-NP---------------------
--PPQPFP----
--------GQGRPQGCSACASKDEED---TMKNLRKTFAGIFGDM

>AplysiacalifornicaSyn2.1_AAK83048
-----------MSFSNFKDSFGSGMNYLRRRFSSGDLQGE--ASDNDDSPNVGGLN----
--------------------------FRK--GPSPSA-----PNSP---------SKSAS
SANLGQRLFSSSSSSSGKPSYNKD----R----CK----TLLVIDDQHTDWSKYFRGKKL
FGDWDVRVEQAEFSELNLAAYSDSGTMVDIQVTRNGTKVV-RSFKPDFVLIRQHVR--DA
HEDWRNLLLGFKYGAIPSVNSLTAEYNFLDKPWVFAQLIEIQKRLGKESFPLIDQAYYPN
HKEML-----ITPKFPVVVKIGHAHSGLGKVRINNHYDFQDLAGVVAVTQG----YSTTE
PFIDAKYDLHIQKIGPSYKAFVRKSISGNWKANTGSAMLEQIAMNERFKLWVDECSQLFG
GLDVVAVEAIHGKDGREHIIEVNGSSMTLLGEAQEEDRRLIAELVLAKMQAMCKPV----

```
---------QTSMSKATSSGAIMHQVNG---------SHSGPQ----AGLRSSH------
-APR--KP--------------------------------------------GQGRGHDG
GPPPQGPMR---APGMPGGPPAPVPR---PRHMN-NP-----------------------
--------------------------------------------------PPQPFP----
--------GQGRPQGCSACASKDEED---TMKNLRKTFAGIFGDM

>AplysiacalifornicaSyn11.1_AAK83047
------------------------MNYLRRRFSSGDLQGE--ASDNDDSPNVGGLN----
--------------------------FRK--GPSPSA-----PNSP---------SKSAS
SANLGQRLFSSSSSSSGKPSYNKD----R----CK----TLLVIDDQHTDWSKYFRGKKL
FGDWDVRVEQAEFSELNLAAYSDSGTMVDIQVTRNGTKVV-RSFKPDFVLIRQHVR--DA
HEDWRNLLLGFKYGAIPSVNSLTAEYNFLDKPWVFAQLIEIQKRLGKESFPLIDQAYYPN
HKEML-----ITPKFPVVVKIGHAHSGLGKVRINNHYDFQDLAGVVAVTQG----YSTTE
PFIDAKYDLHIQKIGPSYKAFVRKSISGNWKANTGSAMLEQIAMNERFKLWVDECSQLFG
GLDVVAVEAIHGKDGREHIIEVNGSSMTLLGEAQEEDRRLIAELVLAKMQAMCKPV----
---------QTSMSKATSSGAIMHQVNG---------SHSGPQ----AGLRSSH------
-APR--KP--------------------------------------------GQGRGHDG
GPPPQGPMR---APGMPGGPPAPVPR---PRHMN-NP-----------------------
--------------------------------------------------PPQPFP----
--------GQGRPQGCSACASKDEED---TMKNLRKTFAGIFGDM

>AplysiacalifornicaSyn7.1_AAK83049
------------------------MNYLRRRFSSGDLQGE--ASDNDDSPNVGGLN----
--------------------------FRK--GPSPSA-----PNSP---------SKSAS
SANLGQRLFSSSSSSSGKPSYNKD----R----CK----TLLVIDDQHTDWSKYFRGKKL
FGDWDVRVEQAEFSELNLAAYSDSGTMVDIQVTRNGTKVV-RSFKPDFVLIRQHVR--DA
HEDWRNLLLGFKYGAIPSVNSLTAEYNFLDKPWVFAQLIEIQKRLGKESFPLIDQAYYPN
HKEMVSNMWLITPKFPVVVKIGHAHSGLGKVRINNHYDFQDLAGVVAVTQG----YSTTE
PFIDAKYDLHIQKIGPSYKAFVRKSISGNWKANTGSAMLEQIAMNERFKLWVDECSQLFG
GLDVVAVEAIHGKDGREHIIEVNGSSMTLLGEAQEEDRRLIAELVLAKMQAMCKPV----
---------QTSMSKATSSGAIMHQVNG---------SHSGPQ----AGLRSSH------
-APR--KP--------------------------------------------GQGRGHDG
GPPPQGPMR---APGMPGGPPAPVPR---PRHMN-NP-----------------------
--------------------------------------------------PPQPFP----
--------GQGRPQGCSACASKDEED---TMKNLRKTFAGIFGDM

>DrosophilamelanogasterSyn_NP_731457.2
--------------------MPPPPAPGQPAGAAPELSLS--FGAGKTPATAAPAP----
---------------------------PR--GVS--A-----PTSP----AKSRESLLQR
VQSLTGAARDQGASILGAAVQSAT---QRAPAFSKDKYFTLLVLDDQNTDWSKYFRGRRL
HGDFDIRVEQAEFRDITVVSSADTGPVVTMAAYRSGTRVA-RSFRPDFVLIRQPPR--DG
SSDYRSTILGLKYGGVPSINSLHSIYQFQDKPWVFSHLLQLQRRLGRDGFPLIEQTFFPN
PRDLFQ-----FTKFPSVLKAGHCHGGVATARLENQSALQDAAGLVSGAGNDSHCYCTIE
PYIDAKFSVHIQKIGNNYKAFMRKSITGNWKTNQGSAMLEQITLTEKYKSWVDEISELFG
GMEVCGLSVVVAKDGREYIISACDSTFALIGDTQEEDRRQIADLVSGRMQNVCRPSMA--
---------QT-GPGKLPSRSSVSSRAES-------PTDEGVAPTPPLPAGPRP------
APMGGPPP-----------------------------------------IPERTSPAVGS
IGRLSSRSSISEVPEEPSSSGPSTVGGVRRDSQTSQS-----------------------
--------------------------------------------------STISSSVSR-
--------AGQRPPQTQNSVVEDAED---TMKNLRKTFAGIFGDM
```
