# Supplementary material for: Synapsins are expressed at neuronal and non-neuronal locations in Octopus vulgaris
Source: Sci Rep. 2019 Oct 28;9:15430. doi: 10.1038/s41598-019-51899-y (PMC6817820; doi:10.1038/s41598-019-51899-y)
Supplement: Supplementary file 5 — Additional file 5 [file 41598_2019_51899_MOESM5_ESM.pdf]

Synapsins are expressed at neuronal and non-neuronal locations in Octopus vulgaris

Federica Maiole[1,2+], Giulia Tedeschi[2,3+], Simona Candiani[4*], Luca Maragliano[1,5], Fabio Benfenati[1,5], Letizia Zullo[1,5*]

Supplementary Info:

Additional file 5: Synapsin probe validation. (a) DIG-labeled RNA synapsin probe loaded on agarose gel. (b) Synapsin mRNA in supraesophageal mass (SEM) is localized at neuronal cell body level. A detail of frontal and sub-frontal lobes is shown (Scale bar, 100 µm).

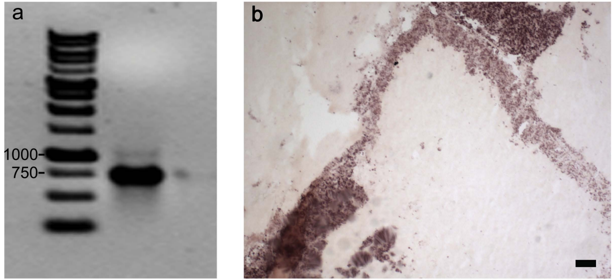
a
b
1000
750
