# Supplementary material for: Synapsins are expressed at neuronal and non-neuronal locations in Octopus vulgaris
Source: Sci Rep. 2019 Oct 28;9:15430. doi: 10.1038/s41598-019-51899-y (PMC6817820; doi:10.1038/s41598-019-51899-y)

Synapsins are expressed at neuronal and non-neuronal locations in Octopus vulgaris

Federica Maiole[1,2+], Giulia Tedeschi[2,3+], Simona Candiani[4*], Luca Maragliano[1,5], Fabio Benfenati[1,5], Letizia Zullo[1,5*]

Supplementary Info:
Additional info on probe length of **additional file.5a**

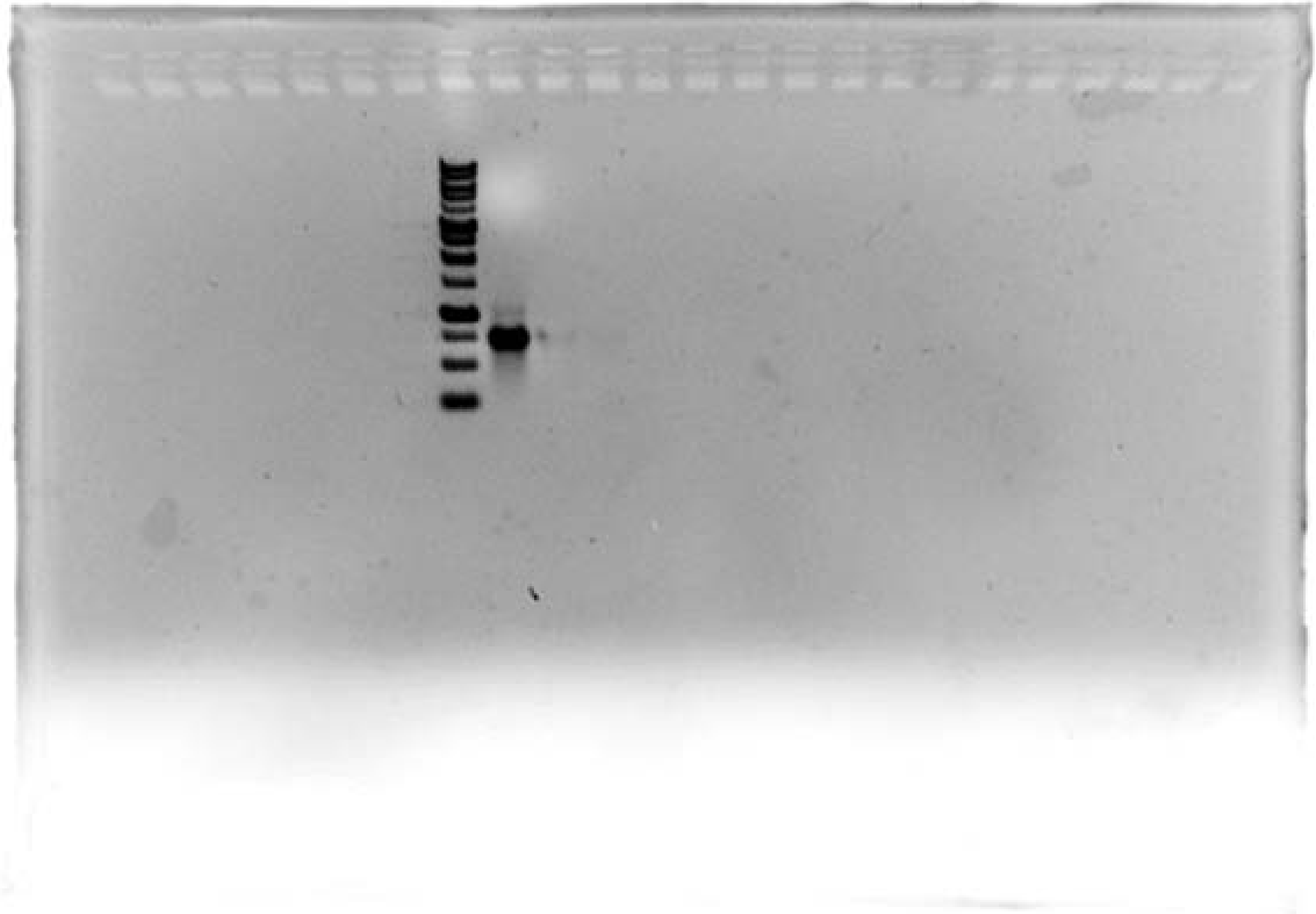

Supplement: Supplementary file 6 — Additional info on probe length of additional file.5a [file 41598_2019_51899_MOESM6_ESM.pdf]
